# Suppressor Mutations in LptF Bypass Essentiality of LptC by Forming a Six-Protein Transenvelope Bridge That Efficiently Transports Lipopolysaccharide

**DOI:** 10.1128/mbio.02202-22

**Published:** 2022-12-21

**Authors:** Federica A. Falchi, Rebecca J. Taylor, Sebastian J. Rowe, Elisabete C. C. M. Moura, Tiago Baeta, Cedric Laguri, Jean-Pierre Simorre, Daniel E. Kahne, Alessandra Polissi, Paola Sperandeo

**Affiliations:** a Dipartimento di Scienze Farmacologiche e Biomolecolari, Università degli Studi di Milano, Milan, Italy; b Department of Chemistry and Chemical Biology, Harvard University, Cambridge, Massachusetts, USA; c University of Grenoble Alpes, CNRS, CEA, IBS, Grenoble, France; e Department of Biological Chemistry and Molecular Pharmacology, Harvard Medical School, Boston, Massachusetts, USA; University of Georgia

**Keywords:** ABC transporter, ATPase, lipopolysaccharide transport, cell envelope, outer membrane biogenesis, proteoliposomes

## Abstract

Lipopolysaccharide (LPS) is an essential component of the outer membrane (OM) of many Gram-negative bacteria, providing a barrier against the entry of toxic molecules. In Escherichia coli, LPS is exported to the cell surface by seven essential proteins (LptA-G) that form a transenvelope complex. At the inner membrane, the ATP-binding cassette (ABC) transporter LptB_2_FG associates with LptC to power LPS extraction from the membrane and transfer to the periplasmic LptA protein, which is in complex with the OM translocon LptDE. LptC interacts both with LptB_2_FG and LptADE to mediate the formation of the transenvelope bridge and regulates the ATPase activity of LptB_2_FG. A genetic screen has previously identified suppressor mutants at a residue (R212) of LptF that are viable in the absence of LptC. Here, we present *in vivo* evidence that the LptF R212G mutant assembles a six-protein transenvelope complex in which LptA mediates interactions with LptF and LptD in the absence of LptC. Furthermore, we present *in vitro* evidence that the mutant LptB_2_FG complexes restore the regulation of ATP hydrolysis as it occurs in the LptB_2_FGC complex to achieve wild-type efficient coupling of ATP hydrolysis and LPS movement. We also show the suppressor mutations restore the wild-type levels of LPS transport both *in vivo* and *in vitro*, but remarkably, without restoring the affinity of the inner membrane complex for LptA. Based on the sensitivity of *lptF* suppressor mutants to selected stress conditions relative to wild-type cells, we show that there are additional regulatory functions of LptF and LptC that had not been identified.

## INTRODUCTION

The hallmark of the cell envelope of Gram-negative bacteria is the presence of an outer membrane (OM) that surrounds the cytoplasmic inner membrane (IM), delimiting an aqueous space where peptidoglycan is embedded (1). The IM consists of a phospholipid bilayer unlike the OM, which is asymmetric, with phospholipids in the inner leaflet and lipopolysaccharide (LPS) in the outer leaflet of its bilayer (2). Tight packing of LPS molecules exclusively in the outer leaflet of the OM creates a barrier that protects Gram-negative bacteria from hydrophobic noxious agents, such as antibiotics, allowing their survival in different and hostile environments (3). Accordingly, LPS is an essential structure in most Gram-negative organisms (4) and defects in the integrity of the LPS layer increase their sensitivity to several antibiotics (3). Therefore, a deeper understanding of the molecular mechanisms that drive the building of the OM is fundamental for the development of novel strategies to fight bacterial infections.

**FIG 1:**
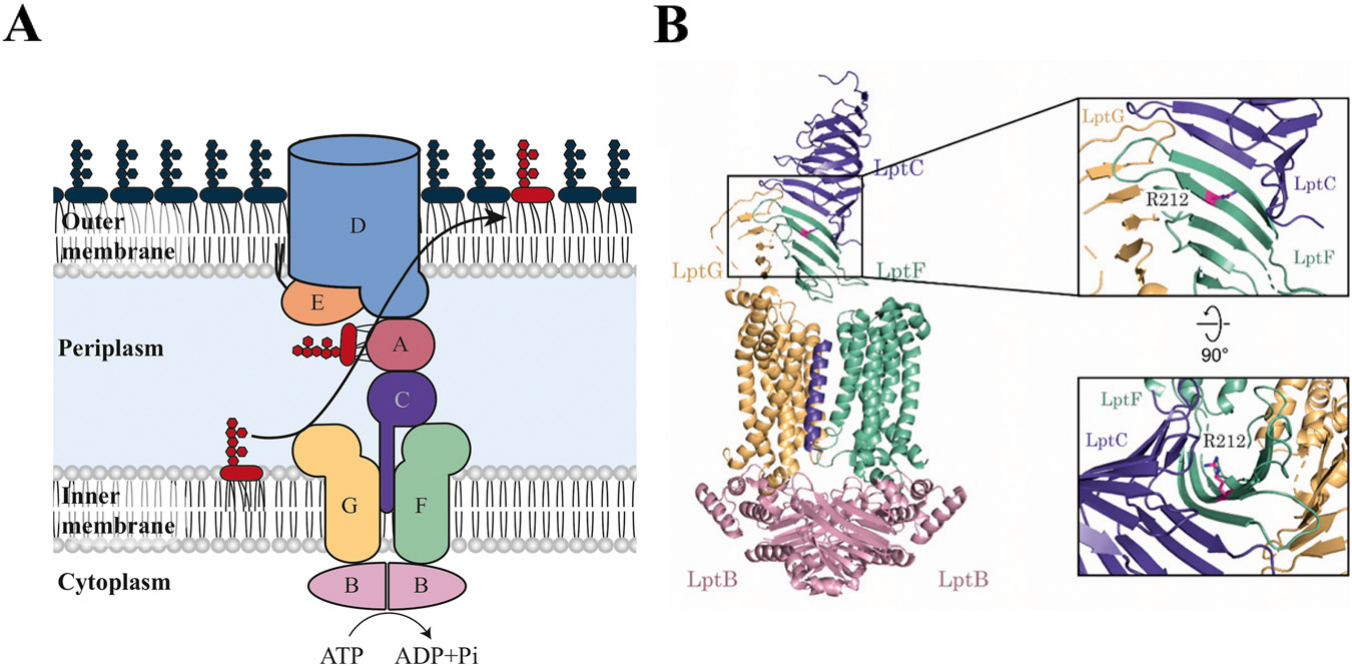
A mutation in LptF bypasses the essentiality of LptC. (A) Schematic representation of the Escherichia coli lipopolysaccharide transport (Lpt) machinery. The lipopolysaccharide (LPS) is transported to the cell surface by a transenvelope complex comprising seven proteins (LptA to G). The ABC transporter LptB_2_FGC extracts LPS from the inner membrane (IM) and, via the soluble domain of LptC, transfers it to LptA. LPS is received at the outer membrane (OM) by the LptDE translocon, which inserts it into the outer leaflet. (B) Ribbon representation of E. cloacae LptB_2_FGC structure (PDB 6MIT). LptC is colored purple, LptG is yellow, LptF is green, and LptB is pink. In the inset, a detail of the junction between the LptF and LptC periplasmic domains is shown. Position R212 is depicted in magenta.

LPS synthesis begins in the cytoplasm and is completed at the periplasmic side of the IM (5). Mature LPS molecules are then extracted from the IM and transported through the periplasm to the OM by the Lpt transenvelope protein complex, composed of seven conserved LPS transport proteins (LptA to G) (Fig. 1A) (6, 7).

In Escherichia coli, the Lpt proteins are essential and organized in three subcomplexes spanning all compartments of the cell (6, 8–11). At the IM, a dimer of the ATP-binding protein LptB is associated with the transmembrane proteins LptF and LptG, and with the bitopic protein LptC, to form the ABC transporter responsible for energizing the detachment of LPS from the IM (8, 10, 12–15). LptB_2_FGC is an unconventional ABC transporter in that the unique transmembrane helix (TM) of LptC is sandwiched between the TMs of LptF and LptG where it contributes to the formation of the cavity that accommodates LPS before its extraction (14, 16, 17). The periplasmic domain of LptC adopts the characteristic β-jellyroll fold shared by the periplasmic domains of five out of seven Lpt proteins (LptACFGD) (16–20) and interacts with the periplasmic domain of LptF, forming a continuous hydrophobic path for LPS export (16).

The energy provided by LptB_2_FGC is employed to move LPS molecules onto LptC (13, 16, 21) and further into the hydrophobic interior of the periplasmic bridge formed by the β-jellyroll domains of LptA (18) and LptD (11, 22–24). The relevance of LptCAD bridge formation has been recently demonstrated by showing that LptA is required *in vitro* to connect vesicles bearing the IM and OM Lpt subcomplexes, thus allowing efficient LPS trafficking (7). Accordingly, each protein of the periplasmic bridges was previously shown to directly interact with LPS (13, 16, 20, 22, 25, 26).

Despite the wealth of structural and biochemical information gained so far, the mechanism that couples energy production by LptB_2_FGC with LPS movement into the transenvelope bridge, as well as the role of the unconventional LptC subunit of the transporter, is still poorly understood. Intriguingly, the interaction of the TM of LptC with LptB_2_FG has been shown to regulate the activity of the transporter, ensuring more efficient coupling between ATP hydrolysis and LPS transport (7, 16, 17, 27). In line with these observations, the topology of LptC is conserved among orthologous proteins, despite the low sequence similarity (28), highlighting the crucial role of this unusual subunit of the Lpt ABC transporter.

To gain deeper insights into the function of LptC in the transporter, we previously screened for suppressor mutations that overcome the lethality of the deletion of *lptC* upon LptA overexpression (29). All suppressor mutants isolated bore substitutions of the residue R212 in the β-jellyroll domain of LptF, strongly suggesting that the mutant LptF protein might enable LptA to substitute for LptC in the assembly of the Lpt machinery. In this work, we use *in vivo* and *in vitro* techniques to understand how these mutations are capable of restoring LPS transport in the absence of LptC.

## RESULTS

### LptF^R212G^ mutant assembles a six-protein Lpt complex.

In a previous work, we showed that three amino acid substitutions at residue R212 of LptF, namely, R212G, R212S, and R212C (collectively defined as *lptF^SupC^* mutants), suppress the lethal phenotype of Δ*lptC* mutations, provided that LptA is overexpressed (29). Among the *lptF^SupC^* mutants, we focused on the *lptF^R212G^* allele because it restores not only cell viability but also OM permeability of Δ*lptC* cells to a nearly wild-type level, albeit retaining sensitivity to novobiocin (29). The localization of R212 in the periplasmic β-jellyroll domain of LptF at the interface with LptC (Fig. 1B) suggests that the suppressor mutants might assemble a functional six-protein Lpt complex (16, 17, 30).

**FIG 2:**
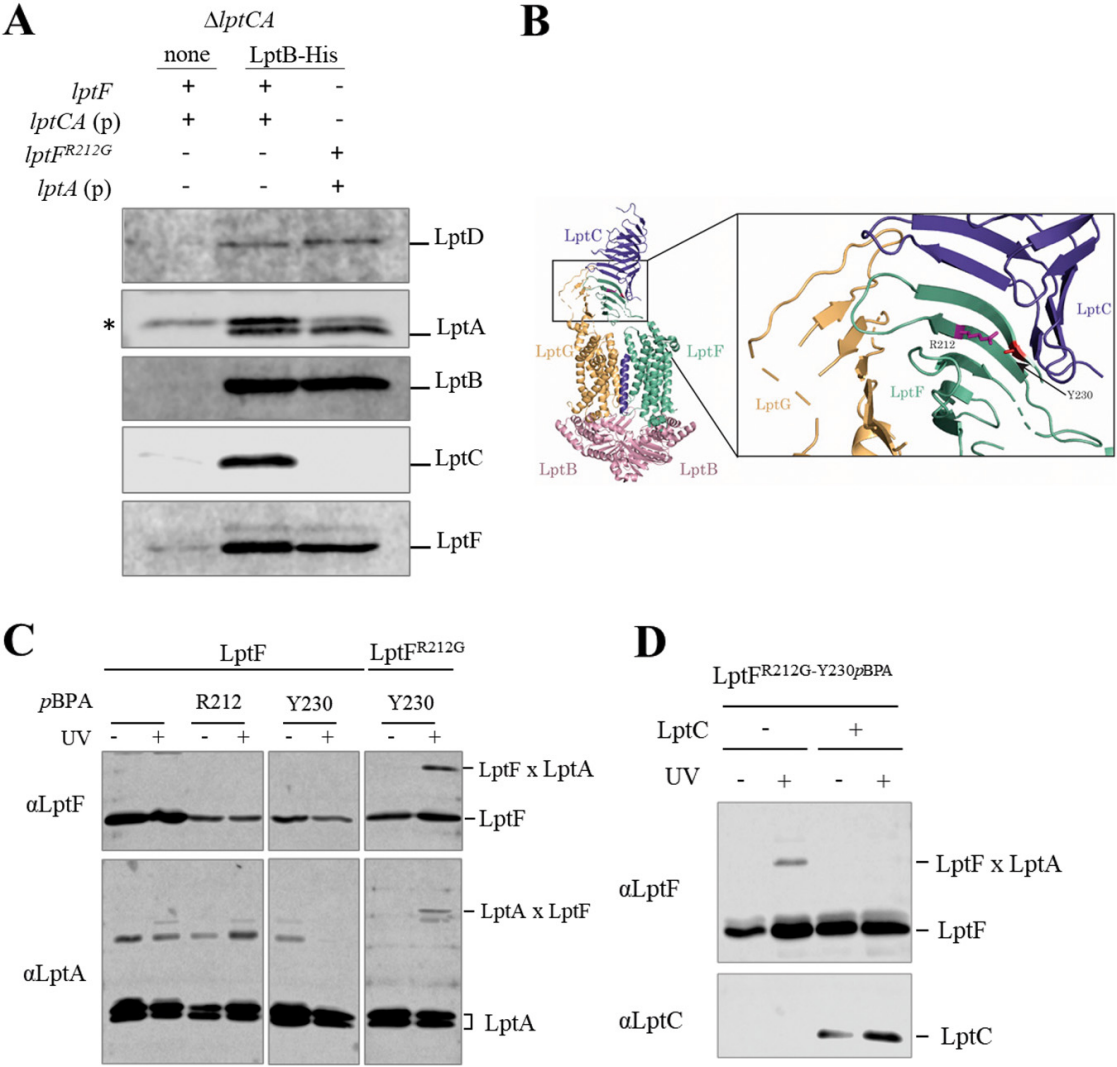
LptBF^R212G^G allows LptF and LptA to directly interact in the absence of LptC. (A) Δ*lptCA* cells (KG286.06 and KG295.01) carrying wild-type or mutant *lptF* alleles (*lptF*, *lptF^R212G,^* respectively) and ectopically expressing *lptA* (p) from pGS321 or *lptCA* (p) from pGS404 were transformed with pET23/42 derived plasmids expressing His tagged *lptB* (LptB-His). Total membranes from an equal number of cells were prepared and subjected to affinity chromatography. Immunoblot analyses with the indicated antibodies are shown. None: void plasmid control. (*) The anti-LptA antibody cross-reacts with an unknown protein that binds nonspecifically to the Ni-NTA resin. Blots are representative of experiments done in triplicate. (B) Ribbon representation of the LptB_2_FGC structure (PDB 6MIT) showing relevant amino acid residues. LptC is colored purple, LptG is yellow, LptF is green, and LptB is pink. Position R212 is depicted in magenta and position Y230 is depicted in red. (C) *In vivo* photo-cross-linking followed by nickel-affinity chromatography from solubilized whole-cell lysate expressing the indicated *p*BPA-containing LptF-His mutants from pET23/42 derived plasmids as bait and LptA from pGS323. UV-dependent photo-cross-linking to LptA was detected with anti-LptA and anti-LptF antibodies. (D) *In vivo* photo-cross-linking followed by nickel-affinity chromatography from solubilized whole-cell lysate expressing LptF^R212G-Y230^*^p^*^PBA^-His (LptF^R212G-Y230^*^p^*^BPA^) and LptA or LptCA from pGS323 and pGS308, respectively. UV-dependent photo-cross-linking to LptA was detected with anti-LptF and LptC antibodies. Blots are representative of experiments done in triplicate.

To assess the assembly of the Lpt complex in the *lptF ^R212G^* mutant in the absence of LptC, an *in vivo* pulldown assay was performed (6). Total membranes were prepared from an equal cell number of *lptC^+^* parental strain (bearing Δ*lptCA* allele complemented by an isopropyl-β-d-1-thiogalactopyranoside (IPTG)-inducible *tacp*-driven copy of *lptC* and *lptA* genes) and the Δ*lptC lptF^R212G^* suppressor mutant (bearing Δ*lptCA* allele complemented by a *tacp*-driven copy of *lptA*), both ectopically expressing C-terminally His-tagged LptB (LptB-His). After *n*-dodecyl-β-d-maltopyranoside (DDM) solubilization, membrane proteins were subjected to affinity chromatography using LptB-His as bait. As a negative control, affinity purification was carried out from solubilized membranes of *lptC^+^* cells transformed with the empty vector. Affinity-purified samples were analyzed by SDS-PAGE followed by immunoblotting with a panel of specific antibodies to assess the copurification of IM and OM Lpt proteins. Notably, in the Δ*lptC lptF^R212G^* suppressor mutant, LptB-His copurified LptA, LptD, and LptF^R212G^, the latter with slightly lower affinity compared to the wild-type strain (Fig. 2A), suggesting that in Δ*lptC lptF^R212G^* mutant the IM and OM Lpt subcomplexes are physically connected. LptC was not enriched in affinity-purified membranes from Δ*lptC lptF^R212G^* cells, and, as expected, *lptC^+^* cells assemble the canonical seven-protein Lpt machinery. It thus appears that the R212G amino acid substitution in LptF can bypass the requirement of LptC and allows the assembly of a six-protein Lpt complex in cells overexpressing LptA. These data suggest that in the Δ*lptC lptF^R212G^* mutant IM and OM are connected by a LptFAD transenvelope bridge.

We previously showed that suppressor mutations in *lptF* are compatible with the presence of LptC and that LptF^SupC^ proteins are functional in a seven-protein Lpt complex (29). Therefore, we assessed whether LptC is recruited in the Lpt complex in *lptF^R212G^* cells. Pulldown from solubilized membranes of *lptC^+^* and Δ*lptC lptF^R212G^* strains, ectopically expressing a C-terminally His-tagged version of LptC (LptC-His), was performed. As a negative control, we used the *lptC^+^* strain transformed with the empty vector. Affinity-purified samples were analyzed as described above. As shown in Fig. S1A in the supplemental material, LptC-His copurified the IM Lpt components LptB and LptF, as well as LptA and the OM Lpt component LptD in both *lptF^+^* and *lptF^R212G^* backgrounds, suggesting that wild-type LptC can be assembled in the Lpt mutant machinery when LptF^R212G^ is present. To rule out the possibility that the formation of an LptB_2_FGC complex occurs through the N-terminal membrane anchor of LptC, LptB_2_FG and LptB_2_F^R212G^G complexes, containing a N-terminal His-tagged LptB, were overexpressed and purified in DDM micelles. The functionality of the purified protein complexes was confirmed by measuring the ATPase activity (Fig. S1B). The proper assembly of the purified complexes was assessed by size exclusion chromatography-multiple angle laser light scattering (SEC-MALLS), a technique that determines the molecular weight and size of heterocomplexes, which confirmed the predicted mass of 134 kDa. When a soluble version of LptC lacking the TM (ΔTM LptC; reference 31) was added to either the wild-type or R212G complexes, an increase in the size of the complex confirmed that the strong interaction of LptC through the β-jellyroll was maintained in the LptB_2_F^R212G^G complex (Fig. S1C). We can therefore conclude that LptF^R212G^ does not impair LptC interaction with the LptB_2_F^R212G^G mutant complex and is compatible with the formation of the canonical seven-protein Lpt complex.

10.1128/mbio.02202-22.5 FIG S1 LptBF^R212G^G does not prevent interaction with LptC. Download FIG S1, PDF file, 0.3 MB. Copyright © 2023 Falchi et al. 2023 Falchi et al. https://creativecommons.org/licenses/by/4.0/ This content is distributed under the terms of the Creative Commons Attribution 4.0 International license.

### LptF^R212G^ directly interacts with LptA in the absence of LptC.

Based on the above results, we postulated that in *lptF^SupC^* cells lacking LptC, the mutant LptB_2_FG complex might interact with LptA, possibly *via* a direct binding of LptF^SupC^ to LptA. To test this hypothesis, we carried out site-specific cross-linking by incorporating the UV-photo-cross-linkable unnatural amino acid *p*-benzoylphenylalanine (*p*BPA) at specific sites in LptF and LptF^R212G^. *p*BPA was incorporated in residues around R212, based on their orientation in the hypothetical interaction interface with LptA (16, 17, 30), and the appearance of UV-dependent cross-linking products was assessed by immunoblotting. We replaced residues F213 and E214, because they are adjacent to the suppressor site R212 on the β7 strand of the β-jellyroll domain of LptF and have different orientations with respect to the hydrophobic cavity; Y230 was replaced since it points toward the interior of the cavity at the C-terminal end of the LptF periplasmic domain (β9 strand). Finally, we selected Q203 since, despite being more distant, it is oriented toward the interior of the cavity, and F160 because it lies on the β2 strand, pointing outwards (Fig. S2 and Fig. 2B). Site-specific mutagenesis of *lptF* and *lptF^R212G^* for *p*BPA incorporation was carried out in pGS445 and in pGS451 plasmids, carrying *lptF^+^* and *lptF^R212G^* alleles, respectively, and expressing LptG and LptAB at comparable levels from the same *tacp* promoter. The mutagenic plasmids were introduced into *lptC^+^ lptF^+^* AM604 strain. All *p*BPA-encoding alleles are functional since they can complement the conditional *araBp-lptFG* mutant NR1113 (10) under nonpermissive conditions (F. A. Falchi and P. Sperandeo, unpublished data). Photo-cross-linking product formation was assessed on whole-cell extracts. As shown in Table 1 and Fig. S3, UV-dependent cross-linking products with a mass compatible with that expected for the LptF-LptA complex were detected by immunoblotting using anti-LptA antibody only in cells expressing LptF^R212G-Y230^*^p^*^BPA^. However, we were unable to clearly detect the LptF-LptA band with anti-LptF antibody, due to its low specificity. Therefore, we sought to increase the concentration of cross-linked LptF-LptA complex in the samples by affinity-purifying LptF after the UV treatment. To this purpose, we used wild-type AM604 cells expressing LptA from a *tacp* promoter and *p*BPA-substituted variants of the C-terminally His-tagged LptF proteins (LptF-His or LptF^R212G^-His) from the leaky expression plasmid pET23/42. Whole-cell lysates from relevant strains were subjected to affinity purification after UV treatment following a previously described procedure (16) and analyzed by immunoblotting using anti-LptA and anti-LptF antibodies. As shown in Fig. 2C, the higher molecular weight band purified by LptF^R212G-Y230^*^p^*^BPA^ was recognized by both the anti-LptA and anti-LptF antibodies, confirming that Y230 residue of LptF^R212G^ is involved in direct interaction with LptA.

**TABLE 1:** Summary of LptF *in vivo* photo-cross-linking in whole-cell experiments

LptF^*a*^	Amber mutation	LptC overexpression^*b*^	XL-LptA
Wild type	None	− +	NO NO
	F160am	− +	NO nt
	Q203am	− +	NO NO
	R212am	− +	NO NO
	E214am	− +	NO NO
	Y230am	− +	NO NO
R212G	None	− +	NO NO
	F160am	− +	NO nt
	Q203am	− +	NO NO
	F213am	− +	NO nt
	E214am	− +	NO NO
	Y230am	− +	YES NO

a LptF variant expressed from a high-copy number plasmid.

b When indicated with +, LptC was expressed from pBAD/HisA vector; nt, not tested.

10.1128/mbio.02202-22.6 FIG S2 Position of residues in LptB_2_FGC substituted with amber codons. Download FIG S2, PDF file, 0.5 MB. Copyright © 2023 Falchi et al. 2023 Falchi et al. https://creativecommons.org/licenses/by/4.0/ This content is distributed under the terms of the Creative Commons Attribution 4.0 International license.

10.1128/mbio.02202-22.7 FIG S3 Mutant LptF^R212G^ interacts with LptA, and the interaction is lost in the presence of ectopically expressed LptC. Download FIG S3, PDF file, 0.5 MB. Copyright © 2023 Falchi et al. 2023 Falchi et al. https://creativecommons.org/licenses/by/4.0/ This content is distributed under the terms of the Creative Commons Attribution 4.0 International license.

We previously observed that reintroduction of a wild-type copy of *lptC* completely restored the OM permeability defects of the *lptF^SupC^* mutants (29). In line with this observation, pulldown experiments show that LptC can be assembled in the Lpt complex expressing LptF^R212G^ (Fig. S1A). We therefore evaluated whether overexpression of LptC affected LptF^R212G^-LptA cross-linking. To this end, we performed UV-photo-cross-linking followed by affinity purification from wild-type AM604 cells expressing LptF^R212G^-His and LptA or LptCA. As shown in Fig. 2D, LptF^R212G^-LptA cross-linking was abolished by LptC overexpression. Interestingly, immunoblotting using anti-LptC antibodies failed to detect a LptC-LptF^R212G^-His cross-linking product, suggesting that residue Y230 is not directly involved in LptF^R212G^-LptC interaction, at least in the presence of LptA. Accordingly, Y230 was not included among the residues mediating LptF-LptC interaction in the wild-type complex (16). These results were confirmed by immunoblotting on UV-photo-cross-linking samples from whole-cell lysates of AM604 cells, ectopically expressing *lptFG-lptAB* and *lptF^R212G^G-lptAB* operons from the *tacp* promoter and LptC from the inducible *araBp* promoter (Table 1 and Fig. S3B). Overall, the experiments described here suggest that, in cells lacking LptC, LptF^R212G^ directly interacts with LptA, but this interaction is different from the one occurring between LptF^R212G^ or LptF and LptC.

### *lptF^SupC^* mutations restore coupling of LPS transport and ATP hydrolysis of LptB_2_FG in the absence of LptC.

**FIG 3:**
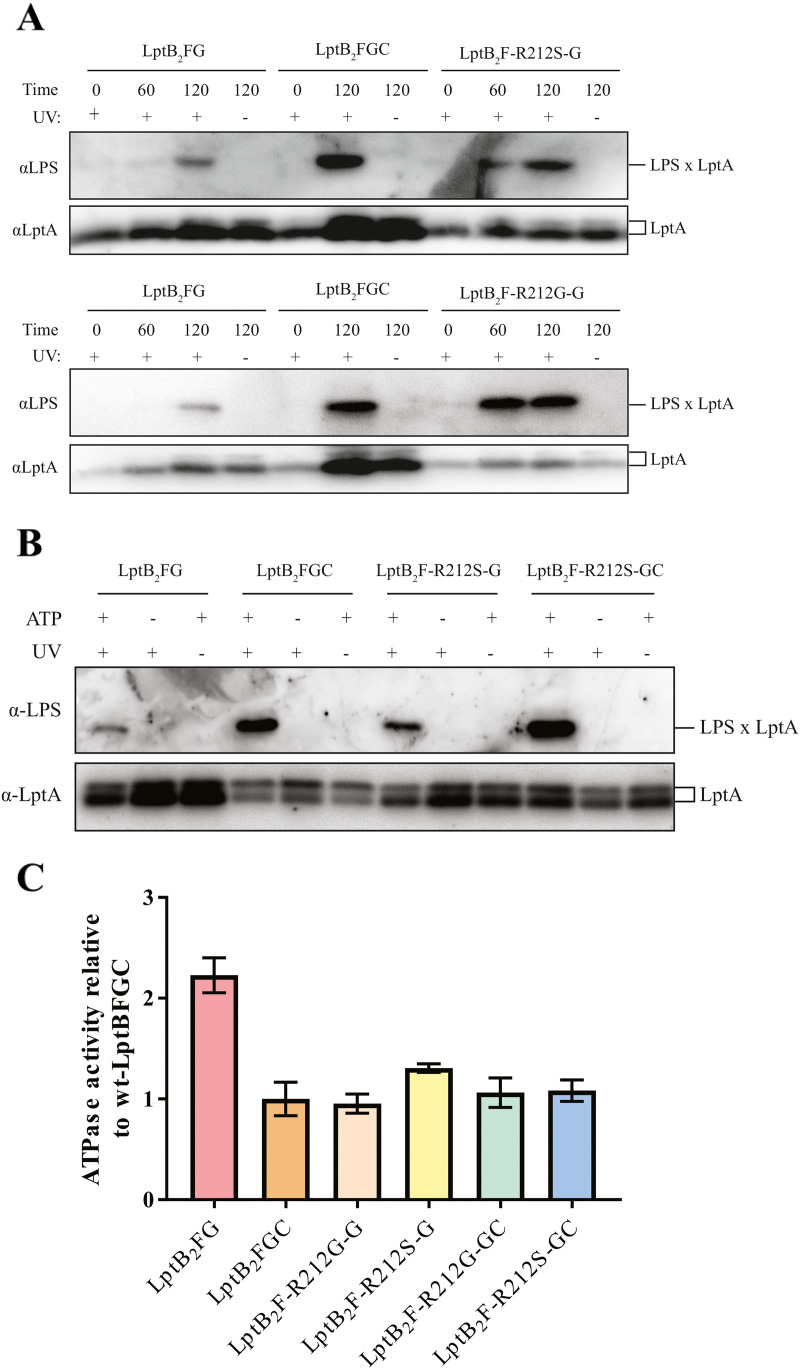
The LptB_2_F^R212G/S^G mutation restores proper coupling of ATP hydrolysis and LPS transport. (A) In a wild-type background, LptB_2_FG, LptB_2_F^R212G^G, or LptB_2_F^R212S^G was overexpressed in cells alongside the overexpression of LptA-I36*p*BPA in all cases and LptC where indicated. *In vivo* transport of LPS was assayed by detecting cross-linking of LPS to position I36 in LptA via Western blotting. Samples were normalized to cell count. Blots are representative of experiments done in triplicate. (B) Proteoliposomes containing the indicated inner membrane complex variants and LPS were incubated with ATP or buffer and LptA-I36pBPA. Samples were incubated for 30 min at 30°C before exposure to UV light as indicated. Blots are representative of experiments done in triplicate. (C) LptB_2_FG, LptB_2_F^R212G^G, and LptB_2_F^R212S^G complexes were purified with and without LptC and reconstituted into proteoliposomes. ATPase activity was assayed by measuring the release of inorganic phosphate over time. Data are normalized to the average of the LptB_2_FGC measurements for 3 to 6 technical replicates per sample.

We wanted to test whether the *lptF^SupC^* mutations also restore LPS transport. To test LPS release to LptA *in vivo*, we generated overexpression strains containing plasmids to allow simultaneous overexpression of LptA^I36^*^p^*^BPA^ and either LptB_2_FG, LptB_2_FGC, LptB_2_F^R212G^G, or LptB_2_F^R212S^G. Aliquots of the cell cultures were either exposed or not exposed to UV light after growing at 37°C for 60 and 120 min after induction. Weak cross-links between LPS and LptA can be observed for the samples overexpressing LptB_2_FG compared to the wild-type LptB_2_FGC as expected (7, 16) (Fig. 3A). Compared to LptB_2_FG, overexpression of LptB_2_F^R212G^G or LptB_2_F^R212S^G restored strong LPS-LptA cross-linking. This suggests that LptB_2_FG complexes containing either LptF^R212G^ or LptF^R212S^ are capable of transferring LPS directly to LptA better than LptB_2_FG. These overall data indicate that *lptF^SupC^* mutations bypass the need for LptC to detach LPS from the IM and transfer it to LptA.

It is known that the TM of LptC modulates the ATP hydrolysis rate of the LptB_2_FG complex to achieve an efficient coupling between LPS transport and ATP hydrolysis (16). We therefore tested the rate of ATP hydrolysis of wild-type and R212G and R212S mutant LptB_2_FG complexes reconstituted in liposomes in the absence or presence of LptC (Fig. 3B and C). As expected, the presence of LptC greatly decreases the rate of ATP hydrolysis of the wild-type LptB_2_FG complex, in agreement with the proposed regulatory role of the TM domain of LptC (16, 17). Interestingly, complexes containing R212G and R212S amino acid substitutions in LptF displayed the same ATPase activity of LptB_2_FGC both in the presence and in the absence of LptC, indicating that the LptF^SupC^ suppressor mutants can fully compensate for the regulatory activity of LptC.

### The *lptF^SupC^* mutations do not increase LptF affinity for LptA.

**FIG 4:**
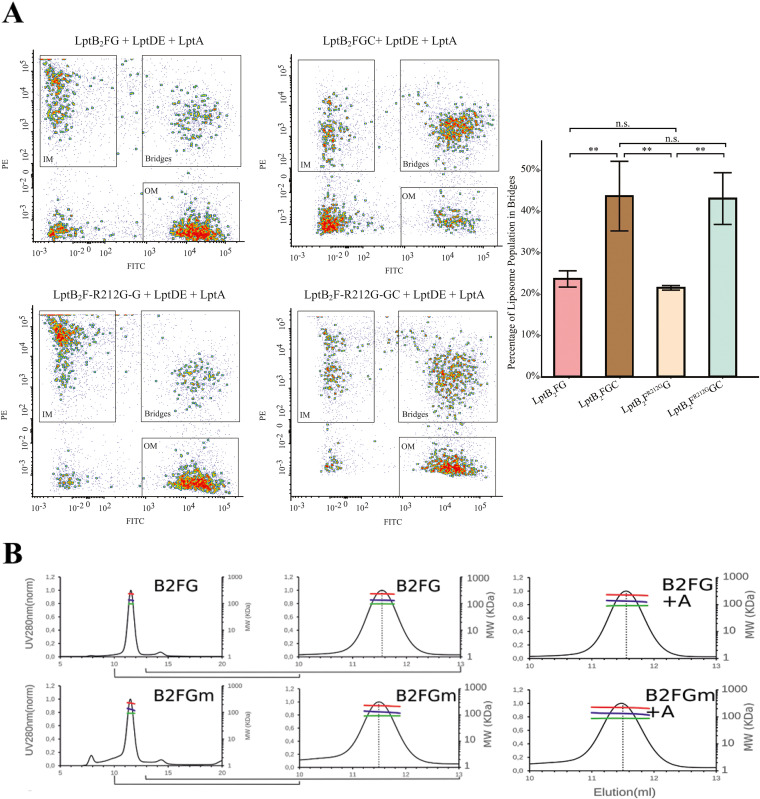
The LptB_2_F^R212G^G mutation facilitates transport of LPS to LptA without increasing the affinity of the LptF-LptA interaction. (A) Fluorescent proteoliposomes containing the indicated inner membrane variant were incubated with fluorescent proteoliposomes containing LptDE and preincubated with LptA. Composition of the proteoliposome mixture was analyzed by flow cytometry in triplicate. 1-tailed *t* tests were used to analyze the difference in bridging populations (**, *P* < 0.05). (B) SEC-MALLS analysis of purified LptB_2_FG (B2FG) and LptB_2_F^R212G^G (B2FGm) in the presence of the monomeric version of LptA (A refers to LptAm, deleted of residues from 160 to 181) did not allow copurification of LptA with any of the complexes, suggesting that the interaction of LptB_2_FG with LptA is much weaker than with LptC. Dotted line indicates the elution volume corresponding to 134 kDa; n.s., not significant.

The overall evidence collected thus far indicated that *lptF^SupC^* enable the formation of a six-protein transenvelope complex and the efficient coupling of LPS transport and ATP hydrolysis in the absence of LptC. However, suppressor mutants display sensitivity to novobiocin, indicative of a partially functional LPS transport (29). To further investigate possible defects in LPS transport that are not restored by *lptF^SupC^* mutations, we sought to reconstitute the capability of LptB_2_FGC complexes to form stable transenvelope bridges in an LptA-dependent manner with LptDE *in vitro* (7). Proteoliposomes containing LptB_2_F^R212G^G had significant (*P* < 0.05) defects in forming stable bridges compared to proteoliposomes containing LptB_2_FGC indicating that LptF^R212G^ is unable to form long-lived bridges *in vitro* (Fig. 4A). Samples with proteoliposomes containing LptB_2_F^R212G^GC were not significantly different from the wild-type LptB_2_FGC complex confirming that the LptF^R212G^ mutant does not change the ability of LptC to complex with LptA and mediate bridge formation. Additionally, there was no significant difference between liposomes containing either LptB_2_F^R212G^G or LptB_2_FG in the ability to form bridges which suggests that the affinity of LptF^R212G^ for LptA is not increased compared to the wild type.

Accordingly, SEC-MALLS analysis of purified LptB_2_FG and LptB_2_F^R212G^G complexes in the presence of the monomeric version of LptA (LptAm, deleted of residues from 160 to 181) (26) did not allow copurification of LptA with any of the complexes (Fig. 4B), consistent with the observation that the interaction of LptF with LptA is much weaker than with LptC, even in the presence of the LptF^R212G^ variant. We thus designed an interaction assay by surface plasmon resonance (SPR). LptB_2_FG and LptB_2_F^R212G^G were covalently immobilized and increasing concentrations of monomeric LptA were flowed over the surface (Fig. S4). A concentration-dependent response was observed, consistent with an interaction with both LptB_2_FG and LptB_2_F^R212G^G. SPR profiles could be fitted at equilibrium and the estimated *K*_d_ values of LptAm are 74 and 81 μM for LptB_2_FG and LptB_2_F^R212G^G, respectively. These values are consistent with the inability to copurify LptA with LptB_2_FG or LptB_2_F^R212G^G by SEC-MALLS. These data suggest that the suppressor mutations restore LPS transfer nearly to wild-type level without significantly increasing the affinity of mutant LptF for LptA.

10.1128/mbio.02202-22.8 FIG S4 Determination of the dissociation constants of the interactions LptB_2_FG-LptAm and LptB_2_F^R212G^G-LptAm by SPR. Download FIG S4, PDF file, 0.2 MB. Copyright © 2023 Falchi et al. 2023 Falchi et al. https://creativecommons.org/licenses/by/4.0/ This content is distributed under the terms of the Creative Commons Attribution 4.0 International license.

10.1128/mbio.02202-22.9 FIG S5 Gating strategy for the collection of the FACS data. Download FIG S5, PDF file, 0.5 MB. Copyright © 2023 Falchi et al. 2023 Falchi et al. https://creativecommons.org/licenses/by/4.0/ This content is distributed under the terms of the Creative Commons Attribution 4.0 International license.

## DISCUSSION

LPS is the major constituent on the surface of the outer membrane, and its presence creates an impermeable barrier that protects bacteria from environmental toxins (3). LPS is synthesized in the cytoplasm and at the IM and therefore must be transported to the cell surface (1). This transport process involves seven essential lipopolysaccharide transport proteins (LptB_2_FGCADE) that form a transenvelope protein bridge (6). Several years ago, a single point mutation in LptF, a component of the inner membrane ATP-binding cassette (LptB_2_FG), was found to bypass the essentiality of LptC (29). This raises the question of why all LPS-containing bacteria have retained LptC (28). LptC is an additional inner membrane component that complexes with LptB_2_FG (15–17, 30). The main known functions of LptC are (i) to coordinate the ATPase activity of the IM complex with the extraction of LPS and its release to the periplasmic LptA (7, 27) and (ii) to mediate a stable formation of a bridge by connecting LptF to LptA (11, 16, 26). In this study, we examined which of the properties of the wild-type Lpt seven-protein complex the LptB_2_F^R212G/S^GADE complex replicates both *in vivo* and *in vitro*. We have found that the regulation of ATPase activity in LptB_2_F^R212G/S^G complexes lacking LptC is similar to the wild-type regulation. The LptF^R212G/S^ mutants transport LPS to LptA and to the cell surface comparably to complexes containing LptC; however, the surprising result is that the LptF^R212G^ mutant has an unstable connection to LptA like wild-type LptF does in the absence of LptC.

We used several techniques to examine the ability of the mutant complex to form and maintain bridges connected directly through an interaction between LptF and LptA. *In vivo* we detected six-protein bridges through both photo-cross-linking and pulldown experiments, suggesting LptA can directly interact with LptF^R212G^ mutant. The viability of *lptF^SupC^* strains depends on the overexpression of LptA (29), and we speculate that high levels of periplasmic LptA can compensate for a low-affinity interaction between LptF^SupC^ and LptA. However, our *in vitro* flow cytometry and SEC-MALLS experiments suggest that the stability of the interaction between LptF^R212G^ and LptA is the same as the interaction between wild-type LptF and LptA. This raises the question of if bridge stability is necessary for sufficient LPS transport to form a proper OM barrier. There are differences between the physiological and *in vitro* systems thatcould account for the differences in LptF^R212G/S^ mutants’ interactions with LptA. In the cell, LptA is usually bound to LptD (reference 6 and P. Sperandeo, unpublished data) and therefore may be better oriented to facilitate interaction with LptF^R212G^, relative to the greater number of orientations of LptD, LptA and LptF^R212G^ available *in vitro*.

On the other hand, a possible explanation for these apparently contrasting observations could be that *in vivo* LptF^R212G/S^ mutants more readily associate with LptA than *in vitro*, possibly due to the active LPS flow. Indeed, although the interaction between LptF^R212G/S^ mutants and LptA is less stable in the absence of LptC, LPS release to LptA and to the cell surface is comparable to wild type (Fig. 3), in line with previous data (29). Therefore, the R212 mutations must facilitate LPS release to LptA without increasing the stability of the *in vitro* interaction between LptF and LptA. R212 lies proximal to a binding site of LPS that exists in wild-type complexes at the interface of the LptF and LptC β-jellyrolls (R223 and Y230 in LptF; T42 and T47 in LptC) (13, 16). LPS must reach this site to be released to LptA (16). Our model is that the interaction of LptF and LptC forms an intermediate binding site, which lowers the energetic barrier for LPS to be released from LptF. The LptF^R212^ mutants may restore the rate of LPS release to LptA by replacing a positively charged amino acid with small uncharged residues and therefore, similarly lowering the energetic barrier. The movement of LPS to this site between LptF^R212^ and LptA could allow LPS to stabilize the Lpt bridge during active LPS transport *in vivo* (32).

Our model predicts that LptF may have an important regulatory function to properly coordinate ATPase activity with LPS transport. LptC has been reported before to have a similar function, therefore this suggests that the wild-type system uses both LptC and LptF to best coordinate efficient LPS transport.

## MATERIALS AND METHODS

### Bacterial strains and growth conditions.

E. coli bacterial strains used in this study are listed in Table S1 in the supplemental material. Unless otherwise stated, bacteria were grown aerobically at 37°C in Luria-Bertani (LB) broth (33) and, when required, 100 μg/mL ampicillin, 30 μg/mL chloramphenicol, 25 μg/mL kanamycin, and 50 μg/mL spectinomycin were added. Solid media were prepared as described above with 1% (wt/vol) agar.

10.1128/mbio.02202-22.2 TABLE S1 Escherichia coli strains used in this study. Download Table S1, DOCX file, 0.01 MB. Copyright © 2023 Falchi et al. 2023 Falchi et al. https://creativecommons.org/licenses/by/4.0/ This content is distributed under the terms of the Creative Commons Attribution 4.0 International license.

### Plasmid construction.

All plasmids are listed in Table S2. To construct any plasmid, the desired gene or DNA fragment was amplified by PCR from the DNA template using primers listed in Table S3. The amplified fragment was digested with appropriate restriction enzymes (New England Biolabs, NEB) and inserted into the same sites of a carrying vector. Amber mutant variants were generated by site-directed mutagenesis using QuikChange II site-directed mutagenesis kit (Agilent Technologies) or Q5 site-directed mutagenesis kit (NEB). All cloned DNA regions obtained by PCR were sequenced to rule out the presence of mutations.

10.1128/mbio.02202-22.3 TABLE S2 Plasmids used in this study. Download Table S2, DOCX file, 0.02 MB. Copyright © 2023 Falchi et al. 2023 Falchi et al. https://creativecommons.org/licenses/by/4.0/ This content is distributed under the terms of the Creative Commons Attribution 4.0 International license.

10.1128/mbio.02202-22.4 TABLE S3 Oligonucleotides used in this study. Download Table S3, DOCX file, 0.02 MB. Copyright © 2023 Falchi et al. 2023 Falchi et al. https://creativecommons.org/licenses/by/4.0/ This content is distributed under the terms of the Creative Commons Attribution 4.0 International license.

### Affinity purification of membrane Lpt complexes for transenvelope bridge assessment.

Membrane Lpt complexes were affinity purified from strains expressing His-tagged LptB from pET23/42-LptB-His plasmid or His-tagged LptC from pET23/42-LptC-His plasmid as described in Chng et al. (6). As a negative control, a strain containing pET23/42 was used.

### *In vivo* UV-photo-cross-linking.

**(i) *In vivo* UV-photo-cross-linking to detect LptF-LptA interaction.** Amber codons were introduced in pET23/42-LptF-His or pET23/42-LptF^R212G^-His plasmids expressing C-terminal His-tagged LptF or LptF^R212G^, respectively, and pSup-BpaRS-6TRN was used to introduce *p*BPA at the specified positions. The assay was performed as described in Reference 16, with minor modifications. Overnight cultures were diluted 1:100 into 200 mL of LB medium supplemented with 0.7 mM *p*BPA (Bachem) and suitable antibiotics and grown to mid-log phase at 30°C. Each culture was split in half, and each sample was pelleted, resuspended in 4 mL of ice-cold PBS, and either used directly or irradiated with UV light at 365 nm for 10 min on ice. All cells were collected and resuspended in 4 mL of ice-cold buffer A (20 mM Tris [pH 7.4], 300 mM NaCl, 5 mM MgCl_2_, and 15 mM imidazole) containing 1% ZW3-14 (*n*-tetradecyl-*N*,*N*-dimethyl-3-ammonio-1-propanesulfonate) (Sigma), 100 μg/mL lysozyme, 1 mM phenylmethanesulfonyl fluoride (PMSF; Sigma), and 50 μg/mL DNase I (Sigma) and lysed by a single cycle through a Cell Disrupter (One Shot Model by Constant Systems Ltd.) at a pressure of 22,000 lb/in^2^ and centrifuged at 4,000 × *g*, 10 min, to remove unbroken cells. Then, nickel affinity purification was performed. A Ni-NTA resin suspension of 0.5 mL (Qiagen) was preequilibrated with 5 mL of buffer A. The mixture was loaded onto the column and allowed to drain by gravity. The column was washed with 1 mL of buffer A and 25 mL of buffer B (20 mM Tris [pH 7.4], 300 mM NaCl, 20 mM imidazole, and 0.1% ZW3-14) and eluted two times with 0.75 mL of buffer C (20 mM Tris [pH 7.4], 300 mM NaCl, 200 mM imidazole, and 0.1% ZW3-14). The eluate was then TCA precipitated (10% wt/vol trichloroacetic acid final) and resuspended in 80 μL of SDS gel-loading buffer for SDS-PAGE and immunoblotting.

**(ii) *In vivo* UV-photo-cross-linking to detect LptA-LPS interaction.** To assess the effect of the R212 mutations on LPS transport, *in vivo* photo-cross-linking experiments were performed as previously described, with minor modifications (13). BL21(DE3) E. coli strains were transformed with pSup-BpaRS-6TRN (34), pET22b-LptA-I36pBPA, and either pCDFDuet-His_6_-LptB_2_FG, pCDFDuet-His_6_-LptB_2_F^R212S^G, or pCDFDuet-His_6_-LptB_2_F^R212G^G. Where indicated, pET22/42-LptC was also transformed. Fifty-milliliter cultures of these strains were grown at 37°C in LB containing 50 μg/mL spectinomycin, 50 μg/mL carbenicillin, 30 μg/mL chloramphenicol, 50 μg/mL kanamycin, and 0.7 mM *p*BPA (Bachem). When the cultures reached an optical density at 600 nm (OD_600_) of ~0.8, expression was induced by the addition of 20 μM IPTG and 0.02% l-arabinose (Sigma). At the indicated time points after inductions, cell counts were measured, and cultures were divided into UV− and UV+ samples; the former was set aside and the latter was exposed to UV light (λ = 365 nm) at room temperature for 10 min.

Samples were purified using Ni-NTA affinity resin to enrich for Western blot detection. Cells were pelleted by centrifugation (5,000 × *g*, 10 min, 4°C), resuspended in 4 mL buffer A (20 mM Tris [pH 8.0], 300 mM NaCl, 15 mM imidazole, 5 mM MgCl2, 1% wt/vol Anzergent 3-14 [Anatrace], and 1 mM PMSF) containing 100 μg/mL lysozyme and 50 μg/mL DNase I and then disrupted by three freeze-thaw cycles alternating between liquid nitrogen and an ice water bath. After the third freeze-thaw cycle, insoluble cell debris was removed by centrifugation for 10 min at 5,000 × *g*. Solubilized supernatants were then applied to Ni-NTA resin (300 μL resin per sample) preequilibrated with buffer A. After flowthrough, the resin was washed three times with 2 mL of wash buffer containing 20 mM Tris (pH 8.0), 300 mM NaCl, 0.05% wt/vol Anzergent 3-14, and 20 mM imidazole. Complexes were eluted three times with 500-μL wash buffer containing 200 mM imidazole. To concentrate the samples, proteins were precipitated from the eluate by the addition of 10% wt/vol trichloroacetic acid, incubated on ice for 30 min, and pelleted at 21,500 × *g* for 30 min at 4°C. After removal of the supernatant, the pellets were resuspended in 1 mL of cold acetone and then pelleted again at 21,500 × *g* for 30 min at 4°C. Finally, the acetone was aspirated off, and the protein pellet was resuspended in approximately 80 μL of SDS gel-loading buffer to allow analysis by Western blotting. The exact amount of resuspension volume was used to normalize samples to the measured cell count before UV exposure.

### Preparation of lipid and LPS stock solutions.

To prepare the lipid stock solution, E. coli polar lipid extract (Avanti Polar Lipids, Inc.) was dissolved in water and sonicated for 30 min to make a 30-mg/mL aqueous suspension stock. The lipid stock solution was flash frozen in liquid nitrogen and stored at −80°C. To prepare a 2 mg/mL aqueous suspension stock of lipopolysaccharide, LPS from E. coli EH100 (Ra mutant; Sigma) was dissolved in water and sonicated for 30 min. The lipopolysaccharide stock solution was flash frozen in liquid nitrogen and stored in aliquots at −80°C.

To prepare the fluorescent lipid stock solutions for flow cytometry, E. coli polar lipid extract (Avanti Polar Lipids, Inc.) was dissolved in chloroform with 1% (molar ratio) ATTO-488 DPPE, ATTO-565 DPPE, or ATTO-647N DPPE (ATTO-TEC GmbH). Lipid mixtures were then dried into a film, hydrated, and lyophilized overnight. The lipid mixtures were then dissolved in water and sonicated for 30 min to make a 30-mg/mL aqueous suspension stock. The lipid stock solution was flash frozen in liquid nitrogen, and stored at −80°C.

### Preparation of proteoliposomes and ATPase assay.

**(i) Overexpression and purification of LptB_2_FG and LptB_2_FGC variants for ATPase assay.** To overexpress His_6_-LptB_2_FG, His_6_-LptB_2_FGC, His_6_-LptB_2_F^R212S^G, His_6_-LptB_2_F^R212S^GC, His_6_-LptB_2_F^R212G^G, and His_6_-LptB_2_F^R212G^GC, C43(DE3), cells were transformed with the relevant variant of pCDFDuet-His_6_-LptB_2_FG. When LptC was co-overexpressed with LptB_2_FG, this strain was also transformed with pET22/42-LptC. Overexpression and purification of each inner membrane complex variant were done as previously reported (21). Cultures were grown at 37°C after diluting overnight cultures 1 to 100 into fresh LB broth supplemented with 50 μg/mL spectinomycin (Sigma), 50 μg/mL carbenicillin (Teknova), 30 μg/mL chloramphenicol (Sigma), and 0.5 mM *p*BPA (Bachem), as appropriate for each inner membrane complex variant. Expression was induced with 300 μM IPTG (Sigma) when the optical density at 600 nm (OD_600_) was ~1, and cultures were grown for 16 h at 18°C. Cells were harvested by centrifugation at 4,200 × *g* for 20 min and resuspended in 50 mM Tris-HCl (pH 7.4), 300 mM NaCl, 2 mM MgCl_2_, supplemented with 1 mM PMSF (Sigma), 100 μg/mL lysozyme (Sigma), and 100 μg/mL DNase I (Sigma). The resuspended cells were passed through an EmulsiFlex-C3 high-pressure cell disruptor three times. The cell lysate was centrifuged at 10,000 × *g* for 10 min to remove unbroken cells. Membranes were isolated by centrifugation at 100,000 × *g* for 1 h. Membranes were resuspended and solubilized in 20 mM Tris-HCl (pH 7.4), 300 mM NaCl, 5 mM MgCl_2_, 10% (vol/vol) glycerol, 1% DDM (Anatrace), and 2 mM ATP at 4°C for 1 h, followed by centrifugation at 100,000 × *g* for 30 min. The supernatant was applied to Ni-NTA Superflow resin (Qiagen) and eluted with 20 mM Tris-HCl (pH 7.4), 300 mM NaCl, 10% (vol/vol) glycerol, 0.05% DDM, and 200 mM imidazole. The eluate was concentrated with an Amicon centrifugation filter, 100-kDa molecular weight cutoff (MWCO; Amicon Ultra; Millipore), and then subjected to size exclusion chromatography on a Superdex 200 10/300 GL column (GE Healthcare) in 20 mM Tris-HCl (pH 7.4), 300 mM NaCl, 5% (vol/vol) glycerol, and 0.05% DDM. Fractions were pooled and concentrated to ~5 mg/mL. All complexes were analyzed by SDS-PAGE to assess purity. Protein was flash frozen in liquid nitrogen and stored at −80°C until use.

**(ii) Preparation of proteoliposomes containing E. coli LptB_2_FG and LptB_2_FGC variants.** Aliquots of lipid stocks and LPS were thawed and sonicated briefly to homogenize. Proteoliposomes containing His_6_-LptB_2_FG, His_6_-LptB_2_FGC, His_6_-LptB_2_F^R212S^G, His_6_-LptB_2_F^R212S^GC, His_6_-LptB_2_F^R212G^G, or His_6_-LptB_2_F^R212S^GC were prepared with and without the addition of LPS by a detergent dilution method (35, 36). Before dilution, a mixture with the following final concentrations was prepared in 20 mM Tris-HCl (pH 8.0), 150 mM NaCl buffer: 7.5 mg/mL lipid stock, 0.5 mg/mL LPS, 0.25% DDM, and 0.86 μM purified inner membrane complex. While making this mixture, the DDM was first added to the lipid stock solution to make detergent-destabilized liposomes. Lipopolysaccharide was added to this mixture, which was subsequently kept on ice for 10 min to allow for mixed detergent-phospholipid-LPS micelles to form. The protein complex was added, and the mixture was left on ice for 20 min. The mixture was then transferred to an ultracentrifuge tube and diluted 100× with cold 20 mM Tris-HCl (pH 8.0) and 150 mM NaCl. After letting the diluted mixture sit on ice for 30 min, the proteoliposomes were pelleted by ultracentrifugation at 300,000 × *g* for 2 h at 4°C. The proteoliposomes were resuspended in 20 mM Tris-HCl (pH 8.0) and 150 mM NaCl and diluted 100× again, then pelleted by ultracentrifugation at 300,000 × *g* for 2 h at 4°C. Finally, for every 100 μL of original mixture (before the first dilution step), 250 μL of cold 20 mM Tris-HCl (pH 8.0), 150 mM NaCl, and 10% glycerol was added. If the resuspended proteoliposomes were not used immediately, they were flash frozen in liquid nitrogen and stored at −80°C. For the preparation of empty proteoliposomes, the purified proteins were substituted with an equal volume of 20 mM Tris-HCl (pH 8.0), 150 mM NaCl, and 10% glycerol.

**(iii) ATPase assays.** ATPase activity was assayed using a modified molybdate method, as previously reported (16). All assays were done in 50 mM Tris-HCl (pH 8.0), 500 mM NaCl, and 10% glycerol (final concentrations). Reactions contained 60% proteoliposomes by volume (prepared as described above, thawed on ice). The remaining volume was composed of Tris-HCl, NaCl, and glycerol, to achieve the final concentrations listed above. Reactions were initiated at 30°C with the addition of ATP-MgCl_2_ (5 mM ATP and 2 mM MgCl_2_). Aliquots were taken at 0, 20, 40, and 60 min and were quenched with an equal volume of 12% SDS (Sigma). Inorganic phosphate was measured using the reported method (37). Absorbance values were measured using a Spectramax Plus 384 plate reader (Molecular Devices). The experiment was repeated three times for each condition. For analysis, linear lines of best fit were calculated for each replicate data set. Plotted data represent the mean value of the slopes and the error bars indicate the standard deviation.

### Overexpression and purification of LptA-I36pBPA.

LptA-I36*p*BPA-His_6_ was overexpressed in the periplasm and purified by making spheroplasts, as previously reported (13). BL21(λDE3) cells with pSup-BpaRS-6TRN and pET22b-LptA-I36*p*BPA-His_6_ were grown to an OD_600_ of ~0.5 in 500 mL LB broth with 50 μg/mL carbenicillin, 30 μg/mL chloramphenicol, and 0.8 mM *p*BPA (Bachem). Protein expression was induced with 50 μM IPTG (Sigma) for 2 h at 37°C. The cells were harvested and converted to spheroplasts. The periplasmic fraction was incubated with Ni-NTA Superflow resin (Qiagen); washed with 20 mM Tris-HCl (pH 8.0), 300 mM NaCl, and 20 mM imidazole; and eluted with 20 mM Tris-HCl (pH 8.0) and 150 mM NaCl, and 200 mM imidazole. The purified LptA-I36*p*BPA-His_6_ was concentrated with 10-kDa cutoff Amicon centrifugal concentrators (Millipore) to ~2.5 mg/mL, flash frozen with liquid nitrogen, and kept at −80°C in 20 mM Tris-HCl (pH 8.0), 150 mM NaCl, and 200 mM imidazole containing 10% glycerol.

### LPS-release assay.

All assays were done in 50 mM Tris-HCl (pH 8.0), 500 mM NaCl, and 10% glycerol (final concentrations). Reactions contained 60% proteoliposomes by volume (prepared as described above, thawed on ice). Proteoliposomes containing LptB_2_FG and LptB_2_FGC variants were incubated with 10× molar excess LptA-I36pBPA at 30°C for 10 min before starting the reaction. The remaining volume was composed of Tris-HCl, NaCl, and glycerol to achieve the final concentrations listed above. Reactions were initiated at 30°C with the addition of ATP–MgCl_2_ (5 mM ATP and 2 mM MgCl_2_). After 30 min, reactions were divided into UV− and UV+ samples; the former set was aside on ice and the latter was exposed to UV light (λ = 365 nm) on ice for 10 min. After this, samples were quenched in an equivalent volume of 12% wt/vol SDS gel-loading buffer to allow analysis by Western blotting.

### SDS-PAGE and immunoblotting.

Homemade 10%, 12.5%, and 15% polyacrylamide gels or 4 to 20% polyacrylamide gradient gels and Tris-glycine running buffer were used for SDS-PAGE/immunoblotting experiments (38). To analyze purified protein complexes or contents of proteoliposomes, homemade Tris-HCl 14% polyacrylamide gels and Tris-glycine running buffer were used followed by staining with Coomassie brilliant blue (Alfa Aesar). For immunoblotting, proteins were transferred onto Immun-Blot PVDF membranes (Bio-Rad), nitrocellulose, or PVDF membranes (Hybond ECL; GE Healthcare). Mouse monoclonal antiserum against the LPS core was purchased from Hycult Biotechnology. Mouse monoclonal anti-His (Sigma-Aldrich) was used at 1:3,000 dilution. Polyclonal sera against LptA (39), LptC and LptD [reference 40 or kindly provided by Shin-ichi Matsuyama (Rikkyo University, Tokyo, Japan)], and LptF (19) (GenScript) were used at dilutions of 1:1,000, 1:3,000, 1:5,000, and 1:10,000, respectively. Additional polyclonal sera against LptA were used in the *in vitro* experiments in Fig. 3 (6). Polyclonal serum anti-LptB (40) (kindly provided by N. Ruiz) was used at a dilution of 1:10,000. As secondary antibodies, anti-rabbit and anti-mouse immunoglobulins (Li-Cor) were used at a dilution of 1:15,000, and bands were detected using an Odissey Fc imaging system (Li-Cor). Alternatively, Lpt proteins were detected by a donkey anti-rabbit horseradish peroxidase (HRP) conjugate secondary antibody (GE Amersham). LPS cross-linking was detected using a sheep anti-mouse HRP conjugate secondary antibody (GE Amersham). Bands were visualized using ECL Prime Western Blotting Detection Reagent (GE Amersham) and imaged using an Azure c600 (Azure Biosystems).

LptF cross-linking was visualized using a goat anti-rabbit IgG HRP-conjugated secondary antibody (Sigma-Aldrich). Filters were developed with the Cyanagen Westar ηC Ultra 2.0 reagent and detected using an Odissey Fc imaging system (Li-Cor).

### Methods for flow cytometry analysis.

**(i) Purification of NusA-His_6_-LptA and preparation of LptA for flow cytometry.** Overexpression and purification of Nus-His_6_-LptA for flow cytometry was done as previously reported (7). In brief, Bl21(λDE3) transformed with pET43.1 Nus-His-LptA were grown at 37°C after diluting overnight cultures 1 to 100 into fresh LB broth supplemented with 50 μg/mL carbenicillin (Teknova). Expression was induced with 50 μM IPTG (Teknova) when the OD_600_ reached ~0.5 and cultures were grown for 16 h at 16°C. Cells were harvested by centrifugation at 4,200 × *g* for 10 min and resuspended in 20 mM Tris-HCl (pH 8.0), 150 mM NaCl, and 10% (vol/vol) glycerol supplemented with a cOmplete protease tablet (Roche), 100 μg/mL lysozyme (Sigma), and 100 μg/mL DNase I (Sigma). The resuspended cells were passed through an EmulsiFlex-C3 high-pressure cell disruptor two times. The cell lysate was centrifuged at 10,000 × *g* for 10 min to remove unbroken cells. Soluble lysate was isolated from membranes by centrifugation at 100,000 × *g* for 30 min. The supernatant was applied to Ni-NTA Superflow resin (Qiagen) and eluted with 20 mM Tris-HCl (pH 8.0), 150 mM NaCl, 10% (vol/vol) glycerol, and 200 mM imidazole. The eluate was concentrated with an Amicon centrifugation filter, 50-kDa MWCO (Amicon Ultra; Millipore). Samples were analyzed by SDS-PAGE to assess purity. Protein was flash frozen in liquid nitrogen and stored at −80°C until use. Thrombin cleavage of NusA-His_6_-LptA: NusA-His_6_-LptA was cleaved with Technical Grade Bovine Thrombin (Biovision) (resuspended in water at 0.9% (vol/vol) NaCl, 0.1% bovine serum albumin (BSA) for 16 h at room temperature (~22°C). NusA-His_6_-LptA was either cleaved immediately after purification, or an aliquot was thawed on ice and cleaved. Cleavage was confirmed by SDS-PAGE analysis. For the cleavage reaction, 0.00396 U enzyme was used per μg NusA-His_6_-LptA. The cleavage mixture was used immediately.

**(ii) Purification of LptD/LptE-His_6_.** Overexpression and purification of LptD/LptE-His_6_ were carried out in a similar manner to the previously reported purification (7). An overnight culture containing C43(DE3) cells transformed with pCOLADuet LptE-His_6_/LptD was diluted 1:100 into 6 1.5 L flasks containing LB supplemented with 0.2% glucose and 30 μg/mL kanamycin. Cultures were grown at 37°C until an OD_600_ of ~0.6 to 0.8 and induced with 500 μM IPTG (Teknova) for 3 h at 37°C. Cells were harvested at 4,200 × *g* for 10 min and resuspended in 20 mM Tris-HCl (pH 8.0), 150 mM NaCl supplemented with a cOmplete protease tablet (Roche), 100 μg/mL lysozyme (Sigma), and 100 μg/mL DNase I (Sigma). The resuspended cells were passed through an EmulsiFlex-C3 high-pressure cell disruptor three times and centrifuged at 10,000 × *g* for 20 min to remove unbroken cells. The supernatant was centrifuged at 100,000 × *g* for 30 min to isolate membranes. The membranes were solubilized in 20 mM Tris HCl (pH 8.0), 150 mM NaCl, 0.5% *N*-lauroylsarcosine sodium salt (Sigma) supplemented with a cOmplete protease tablet (Roche) at 4°C for 2 h and then centrifuged for 30 min at 100,000 × *g*. The resulting membrane pellet was resuspended in 20 mM Tris-HCl (pH 8.0) and 150 mM NaCl and centrifuged for 30 min at 100,000 × *g* to remove the remaining *N*-lauroylsarcosine sodium salt. The washed pellet was solubilized in 20 mM Tris-HCl (pH 8.0), 300 mM NaCl, 20 mM imidazole, and 1.0% Anzergent 3-14 (Anatrace) at 4°C for 2 h and then centrifuged at 100,000 × *g* for 1 h. The supernatant was applied to Ni-NTA Superflow (Qiagen), and the flowthrough was collected and reapplied to the column. The column was washed with 20 column volumes of 20 mM Tris-HCl (pH 8.0), 300 mM NaCl, 20 mM imidazole, and 1.0% Anzergent 3-14 (Anatrace) followed by 20 column volumes of 20 mM Tris-HCl (pH 8.0), 300 mM NaCl, 20 mM imidazole, and 1.0% *n*-octylglucoside (OG; Anatrace). The column was eluted with 6 column volumes of 20 mM Tris-HCl (pH 8.0), 300 mM NaCl, 200 mM imidazole, and 1.0% OG (Anatrace). The eluate was concentrated with an Amicon centrifugation filter, 100-kDa MWCO (Amicon Ultra; Millipore). The concentrated protein was centrifuged at 15,000 × *g* for 10 min to remove aggregate protein and then injected onto a Superdex 200 Increase 10/300 GL column (GE Healthcare) for size exclusion chromatography in 20 mM Tris-HCl (pH 8.0), 150 mM NaCl, and 1.0% OG. Fractions were pooled and concentrated on another Amicon centrifugation filter, 100-kDa MWCO, until reaching a concentration of ~10 mg/mL. Purified complexes were analyzed by SDS-PAGE to ensure purity. Samples were flash frozen in liquid nitrogen and stored at −80°C until use.

**(iii) Preparation of proteoliposomes containing E. coli LptB_2_FG and LptB_2_FGC variants for flow cytometry.** Aliquots of fluorescent lipid stocks and LPS were thawed and sonicated briefly to homogenize. Proteoliposomes containing His_6_-LptB_2_FG, His_6_-LptB_2_FGC, His_6_-LptB_2_F^R212S^G, His_6_-LptB_2_F^R212S^GC, His_6_-LptB_2_F^R212G^G, or His_6_-LptB_2_F^R212S^GC, were prepared by a detergent dilution method (35, 36). Before dilution, a mixture with the following final concentrations was prepared in 20 mM Tris pH 8.0, 150 mM NaCl buffer: 3.25 mg/mL ATTO-565 lipid stock, 3.25 mg/mL ATTO-647N lipid stock, 2 mg/mL Ra-LPS, 0.05% DDM, and 0.86 μM purified inner membrane complex. While making this mixture, the DDM was first added to the lipid stock solution to make detergent-destabilized liposomes. Lipopolysaccharide was added to this mixture, which was subsequently sonicated in a chilled water bath for 1 min and then kept on ice for 10 min to allow for mixed detergent-phospholipid-LPS micelles to form. The protein complex was added, and the mixture was left on ice for 20 min. The mixture was then transferred to an ultracentrifuge tube and diluted 100× with cold 20 mM Tris-HCl (pH 8.0) and 150 mM NaCl. After letting the diluted mixture sit on ice for 30 min, the proteoliposomes were pelleted by ultracentrifugation at 300,000 × *g* for 2 h at 4°C. For every 100 μL original mixture (before the dilution step), 250 μL of cold 20 mM Tris-HCl (pH 8.0), 150 mM NaCl, and 10% glycerol were added. If the resuspended proteoliposomes were not used immediately, they were flash frozen in liquid nitrogen and stored at −80°C.

**(iv) Preparation of proteoliposomes containing E. coli LptDE for flow cytometry.** Proteoliposomes containing LptDE-His_6_ were prepared by a detergent dilution method (35, 36). Before dilution, a mixture with the following final concentrations was prepared in 20 mM Tris pH 8.0, and150 mM NaCl buffer: 4.5 mg/mL ATTO-488 lipid stock, 4.5 mg/mL ATTO-647N lipid stock, 1.5% OG, and 1.5 μM purified inner membrane complex. While making this mixture, the OG was first added to the lipid stock solution and subsequently sonicated in a chilled water bath for 1 min before being kept on ice for 10 min to allow for mixed-detergent-phospholipid micelles to form. The protein complex was added, and the mixture was left on ice for 20 min. The mixture was then transferred to an ultracentrifuge tube and diluted 100× with cold 20 mM Tris-HCl (pH 8.0) and 150 mM NaCl. After letting the diluted mixture sit on ice for 30 min, the proteoliposomes were pelleted by ultracentrifugation at 300,000 × *g* for 2 h at 4°C. For every 100 μL original mixture (before the dilution step), 200 μL of cold 20 mM Tris-HCl (pH 8.0), 150 mM NaCl, and 10% glycerol was added. If the resuspended proteoliposomes were not used immediately, they were flash frozen in liquid nitrogen and stored at −80°C. Before use in flow cytometry, fluorescent proteoliposomes containing LptDE were incubated with either 10-fold molar excess LptA or NusA-His_6_-LptA for 30 min in 20 mM Tris-HCl (pH 8.0), 150 mM NaCl, and 10% glycerol. Proteoliposomes were then recovered by ultracentrifugation at 300,000 × *g* for 1 h at 4°C and resuspended in their original volume in 20 mM Tris-HCl (pH 8.0), 150 mM NaCl, and 10% glycerol.

### Flow cytometry analysis of proteoliposomes containing E. coli LptB_2_FG and LptB_2_FGC variants.

Samples for flow cytometry were made before dilution as follows in 20 mM Tris-HCl (pH 8.0), 150 mM NaCl, and 10% glycerol: 75 nM LptDE associated with the indicated variant of LptA in ATTO-488/ATTO-647N proteoliposomes and 34 nM LptB_2_FGC (or indicated inner membrane variant). Mixtures were allowed to equilibrate at room temperature for at least 2 min before being diluted 20-fold in 20 mM Tris-HCl (pH 8.0), 150 mM NaCl, and 10% glycerol.

Flow cytometric studies were performed on a BD FACSAria flow cytometer in a manner similar to a previously reported method (7). All drops were excited on a 640-nm excitation laser source, bandpass filter 660/20 nm, and PMT set at 400V for ATTO-647N detection. Only drops above a fluorescence threshold of 1,000 on this detection were recorded to remove nonliposomal particles. The forward scatter (FSC) and side scatter (SSC) thresholds were both set as 200. Samples were excited on a 488-nm excitation laser source, band-pass filter 530/15 nm for ATTO-488 detection, and excited on a 561-nm excitation laser source, band-pass filter 586/15 nm for ATTO-565 detection. Compensation was conducted in FACSDivaTM software using ATTO-488 outer membrane proteoliposomes and ATTO-565 inner membrane proteoliposomes as single stain controls. Compensation values were set to 0.2% of the 488-nm laser in the 565-nm detection and 0.2% of the 565-nm laser in the 488-nm detection. 10,000 particle counts were recorded for each replicate. Three technical replicates of liposome preparations were analyzed for each variant of LptB_2_FG and LptB_2_FGC. Samples were gated as indicated in Fig. S5. In brief, samples were gated by FSC-A (10 to 3,000), 640 nm-excitation (APC-A) (1,000 to 80,000), SSC-A (200 to 8,000), and SSC-W (50 to 100). Liposomes registering a 561-nm excitation/emission value above 100 and a 488-nm excitation/emission value above 1,000 were gated as bridged liposomes. One-tailed *t* tests were conducted to determine if samples were significantly worse at bridging than the LptB_2_FGC control. Representative plots for the publication were generated in FCSExpress software and statistical analysis and graphing were done in R.

### SEC-MALLS analysis.

**(i) Purification of LptB_2_FG and LptB_2_F^R212G^G complexes.** To overexpress and purify the wild-type and mutant IM Lpt complexes, *E coli* KRX cells were transformed with pCDFDuet vector expressing N-terminal His-tagged LptB, LptF, and LptG (pCDFDuet-His_6_-LptBFG) or N-terminal His-tagged LptB, LptF^R212G^ and LptG (pCDFDuet-His_6_-LptBF^R212G^G). Cultures were grown in 1 L of LB medium to an OD_600_ of 0.8 at 37°C. Expression was induced with 0.02% l-rhamnose and 0.02% arabinose for 3 h at 37°C. Cells were harvested by centrifugation at 4°C at 5,000 × *g* for 20 min and stored at −80°C. The cell pellets were resuspended in Lysis buffer (50 mM Tris HCl [pH 7.4], 300 mM NaCl, and 1 mM MgCl_2_) supplemented with 1 mM PMSF (Sigma), 100 μg/mL lysozyme, and 50 μg/mL DNase I. The cells were disrupted by a single passage through a cell disrupter (One Shot model; Constant Systems Ltd.) at a pressure of 22,000 lb/in^2^. Unbroken cells were removed by centrifugation at 4,500 × *g* for 15 min and membranes were isolated by ultracentrifugation at 100,000 × *g* for 1 h. Membranes were resuspended and solubilized in 20 mL of Resuspension buffer [20 mM Tris-HCl (pH 7.4), 300 mM NaCl, 5 mM MgCl_2_, 10% (vol/vol) glycerol, 1% (wt/vol) DDM (Sigma), and 2 mM ATP] at 4°C for 1 h, followed by centrifugation at 100,000 × *g* for 30 min. The supernatant was supplied with 2 mM imidazole and rocked for 1 h with 1 mL of TALON metal affinity resin (Clontech), preequilibrated with Affinity buffer [20 mM Tris-HCl (pH 7.4), 300 mM NaCl, 5 mM MgCl_2_, 10% (vol/vol) glycerol, and 0.05% (wt/vol) DDM]. The column was washed with 20 column volumes (cv) of Affinity buffer plus 2 mM imidazole and 10 cv of Affinity buffer plus 10 mM imidazole. Proteins were eluted with 8 cv of affinity buffer plus 100 mM imidazole and the eluate was concentrated up to 2 mL with an Amicon centrifugation filter, 10-kDa MWCO (Amicon Ultra; Millipore). The buffer was changed to Exchange buffer (20 mM Tris-HCl [pH 7.4], 300 mM NaCl, and 0.05% [wt/vol] DDM) and the sample was concentrated up to 0.4 mL as above. The protein concentration was determined by Bradford assay (Thermo Fisher) using BSA as a standard and samples were loaded on SDS-PAGE and Coomassie stained. For the purification of the LptB_2_FG complex, an additional step of size exclusion chromatography was performed before determining protein concentration. The LptB_2_FG concentrated eluate was applied to a HiLoad 16/600 Superdex 200 gel filtration column preequilibrated with 20 mM Tris-HCl pH 7.4, 300 mM NaCl, and 0.05% (wt/vol) DDM. Peak fractions were combined and concentrated with an Amicon 100-kDa MWCO centrifugation filter (Amicon Ultra; Millipore).

**(ii) Purification of ΔTM LptC (LptCΔ1-23) and LptAm (LptAΔ160-181).** E. coli LptC lacking the first 23 residues of the transmembrane domain was expressed from a plasmid (LptC pQESH) with an N-terminal His-Tag and purified as described by Baeta et al. (41).

LptAm coding for residues 1 to 159 followed by a SGRVEHHHHHH TAG in a pET21b-derived vector was expressed and purified as described by Laguri et al. (26).

**(iii) SEC-MALLS.** LptB_2_FG and LptB_2_F^R212G^G purified in DDM were injected (40 μL volume at 8 μM) alone or in the presence of 50 μM ΔTM LptC or LptAm in 20 mM Tris, 150 mM NaCl, and 0.05% DDM buffer at 25°C on a Superdex S200 (10/300GL) connected to an HPLC-Multi Angle light scattering (DynaPro Nanostar), refraction index (Optilab rex), and Optical density detectors (SPD-M20A). Data analysis is performed with ASTRA 5.4.3.20 software (WYATT). Two-component analysis with the protein conjugate method was used for the determination of DDM micelle and protein complexes masses.

### Other methods.

*In vivo* site-specific UV-photo-cross-linking and whole-cell lysate analysis for LptF-LptA interaction, ATPase activity in DDM micelles and SPR-based binding assays are described in detail in Text S1 in the supplemental material.

10.1128/mbio.02202-22.1 TEXT S1 Supplemental methods. Download Text S1, PDF file, 0.09 MB. Copyright © 2023 Falchi et al. 2023 Falchi et al. https://creativecommons.org/licenses/by/4.0/ This content is distributed under the terms of the Creative Commons Attribution 4.0 International license.
