# Supplementary material for: Suppressor Mutations in LptF Bypass Essentiality of LptC by Forming a Six-Protein Transenvelope Bridge That Efficiently Transports Lipopolysaccharide
Source: mBio. 2022 Dec 21;14(1):e02202-22. doi: 10.1128/mbio.02202-22 (PMC9972910; doi:10.1128/mbio.02202-22)
Supplement: FIG S1 [file mbio.02202-22-s0005.pdf]

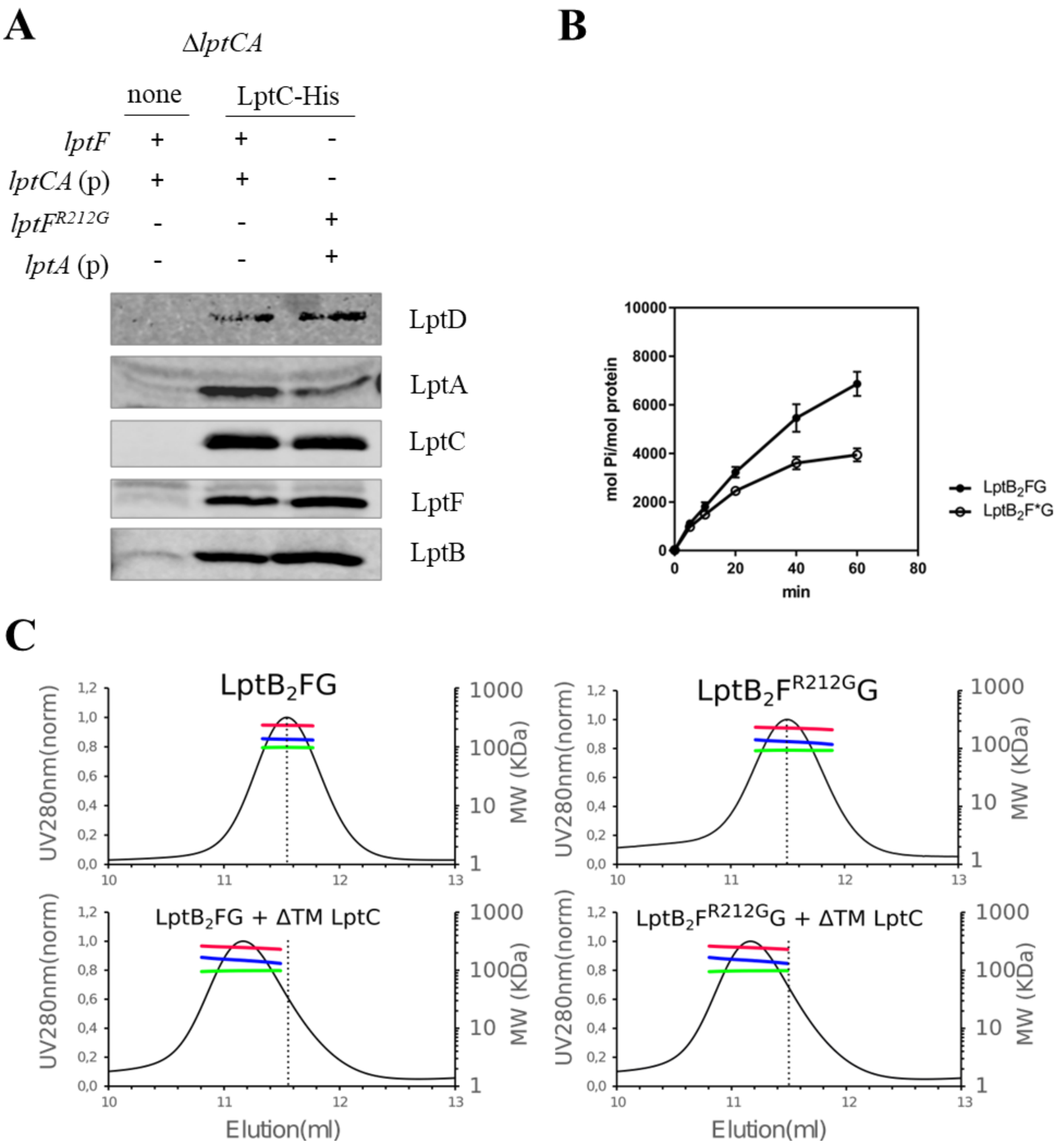

Figure S1. LptBF^{R212G}G does not prevent interaction with LptC. (A) Δ*lptCA* cells (KG286.06 and KG295.01) carrying wild type or mutant *lptF* alleles (*lptF*, *lptF^{R212G}* respectively) and ectopically expressing *lptA* (p) from pGS321 or *lptCA* (p) from pGS404 were transformed with pET23/42 derived plasmids expressing His tagged LptC (LptC-His). Total membranes from an equal number of cells were prepared and subjected to affinity chromatography. Immunoblot analyses with the indicated antibodies are shown. None: void plasmid control. (B) ATPase activity of LptB$_2$FG and LptB$_2$F^{R212G}G (LptB$_2$F*G) in DDM. Each point represents mean ± s.d. of three separate measurements. (C) SEC MALLS elution profiles of DDM-purified LpB$_2$FG and LpB$_2$F^{R212G}G complexes alone (upper panels) and 1:1 mixture of LpB$_2$FG: ΔTM LptC and LptB$_2$F^{R212G}G: ΔTM LptC (lower panels). Dotted line indicates the elution volume corresponding to 134 kDa.
