# Supplementary material for: Suppressor Mutations in LptF Bypass Essentiality of LptC by Forming a Six-Protein Transenvelope Bridge That Efficiently Transports Lipopolysaccharide
Source: mBio. 2022 Dec 21;14(1):e02202-22. doi: 10.1128/mbio.02202-22 (PMC9972910; doi:10.1128/mbio.02202-22)
Supplement: FIG S2 [file mbio.02202-22-s0006.pdf]

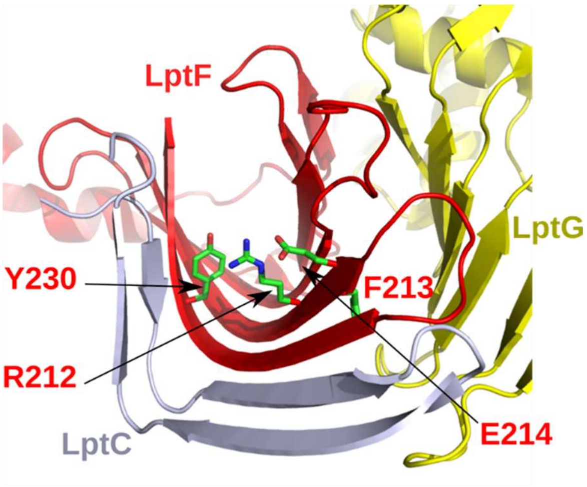

Figure S2. Position of residues in LptB$_2$FGC substituted with amber codons. Ribbon representation of a detail of LptF periplasmic domain in *E. cloacae* LptB$_2$FGC structure (PDB 6MIT). LptC is coloured purple, LptF red and LptG yellow. Residues are depicted as sticks.
