# Supplementary material for: Suppressor Mutations in LptF Bypass Essentiality of LptC by Forming a Six-Protein Transenvelope Bridge That Efficiently Transports Lipopolysaccharide
Source: mBio. 2022 Dec 21;14(1):e02202-22. doi: 10.1128/mbio.02202-22 (PMC9972910; doi:10.1128/mbio.02202-22)
Supplement: FIG S3 [file mbio.02202-22-s0007.pdf]

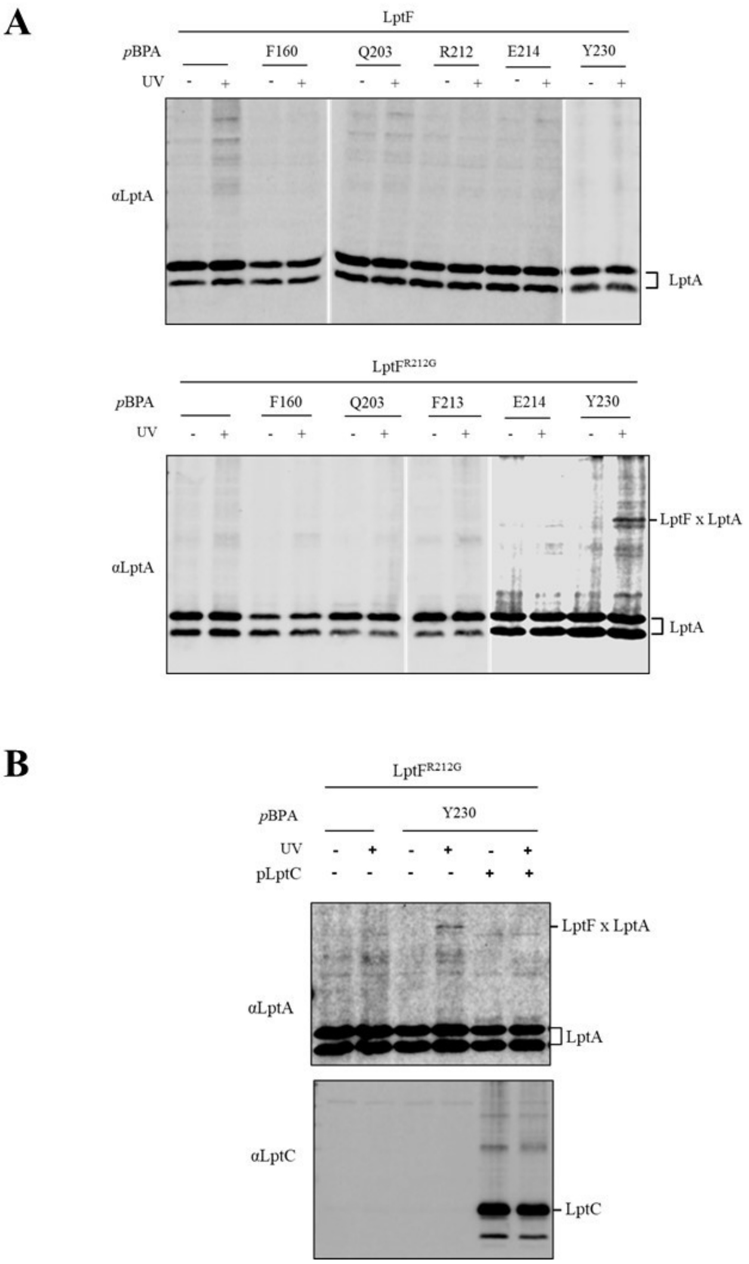

Figure S3. Mutant LptF^{R212G} interacts with LptA and interaction is lost in the presence of ectopically expressed LptC. (A) Specific amino acid positions in LptF and LptF^{R212G} were mutated to incorporate *p*BPA. Amber codons were introduced into *lptF* gene in pGS445 and pGS451 plasmids, harbouring *lptFGAB* and *lptF*^{R212G}*GAB*, respectively. (B) To assess the effect of LptC overexpression, cells were transformed with plasmid pBAD/HisA-LptC (pLptC) expressing LptC from the inducible *araBp* promoter. Crosslinking products were detected in TCA precipitated whole cell extracts separated onto SDS-PAGE followed by immunoblotting with anti-LptA or anti-LptC antibodies. Only residue Y230 in mutant LptF^{R212G} protein crosslinked to LptA upon UV-irradiation and this interaction was abolished by LptC overexpression.
