# Supplementary material for: Suppressor Mutations in LptF Bypass Essentiality of LptC by Forming a Six-Protein Transenvelope Bridge That Efficiently Transports Lipopolysaccharide
Source: mBio. 2022 Dec 21;14(1):e02202-22. doi: 10.1128/mbio.02202-22 (PMC9972910; doi:10.1128/mbio.02202-22)
Supplement: FIG S4 [file mbio.02202-22-s0008.pdf]

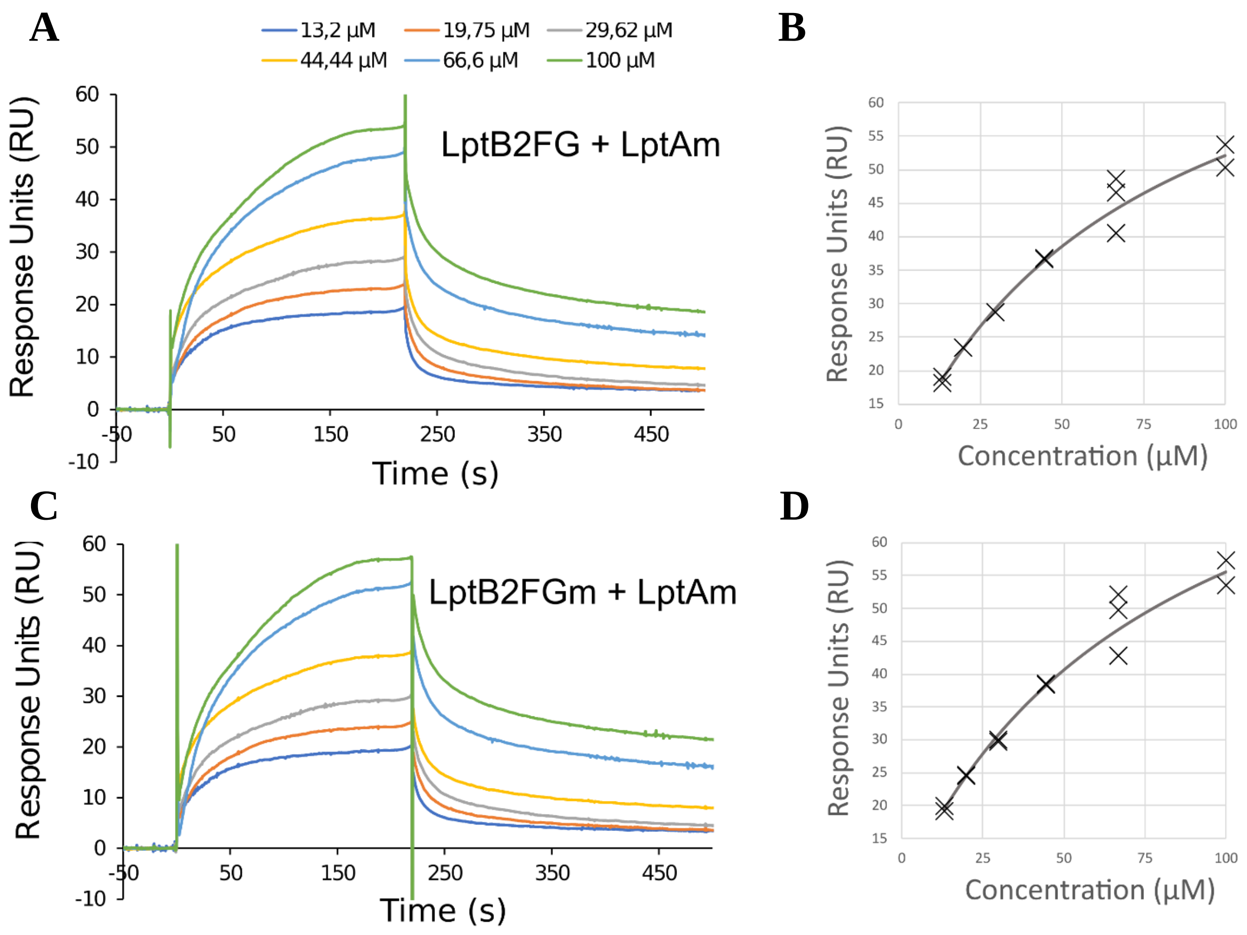

Figure S4. Determination of the dissociation constants of the interactions $LptB_2FG$-LptAm and $LptB_2F^{R212G}G$-LptAm by SPR. Sensorgrams of LptAm (LptA monomeric version deleted of residues from 160 to 181) injected at increasing concentrations over immobilized $LptB_2FG$ (A) and $LptB_2F^{R212G}G$ ($LptB_2FGm$) (C). Steady-state analysis of $LptB_2FG$-LptAm (B) and $LptB_2F^{R212G}G$-LptAm (D) interactions.
