# Supplementary material for: Suppressor Mutations in LptF Bypass Essentiality of LptC by Forming a Six-Protein Transenvelope Bridge That Efficiently Transports Lipopolysaccharide
Source: mBio. 2022 Dec 21;14(1):e02202-22. doi: 10.1128/mbio.02202-22 (PMC9972910; doi:10.1128/mbio.02202-22)
Supplement: FIG S5 [file mbio.02202-22-s0009.pdf]

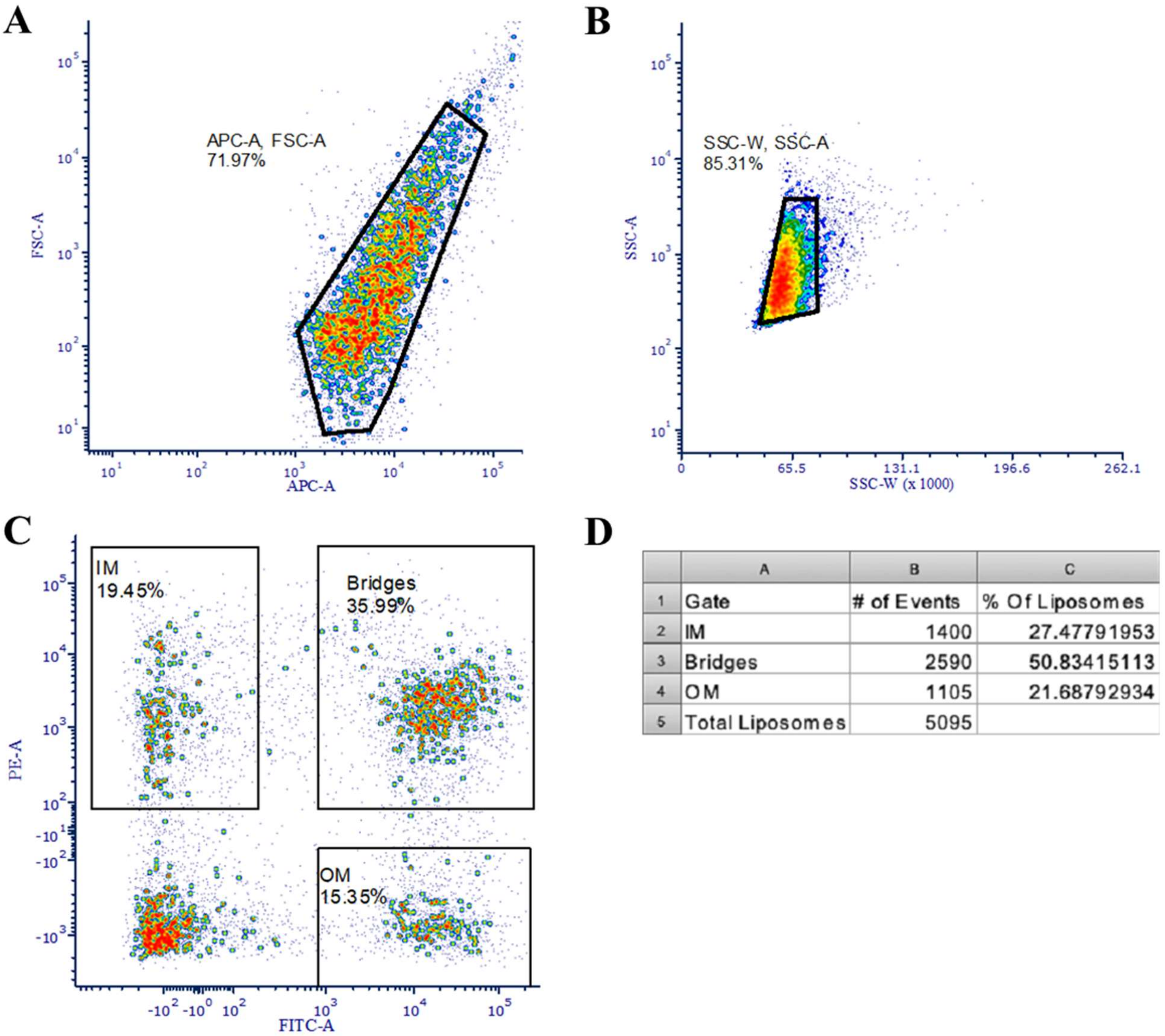

	A	B	C
1	Gate	# of Events	% Of Liposomes
2	IM	1400	27.47791953
3	Bridges	2590	50.83415113
4	OM	1105	21.68792934
5	Total Liposomes	5095	

Figure S5. Gating strategy for the collection of the FACS data. The gating method used is described in depth in the methods section but in brief: (A) Droplets were gated for sufficient FSC-A size and APC-A fluorescence to ensure they contained liposomes. (B) Events from (A) were gated to exclude aggregates as determined by SSC-A and SSC-W. (C) Events from (B) were then plotted for PE and FITC fluorescence. Events representing bridges were determined by events reaching the minimum FITC-A threshold for outer membrane complex containing liposomes and the minimum PE-A threshold for the inner membrane complex containing liposomes. (D) Data was initially collected as number of events in each gate and percent of events in the bridged gate compared to all liposomes was calculated.
