# Supplementary material for: Suppressor Mutations in LptF Bypass Essentiality of LptC by Forming a Six-Protein Transenvelope Bridge That Efficiently Transports Lipopolysaccharide
Source: mBio. 2022 Dec 21;14(1):e02202-22. doi: 10.1128/mbio.02202-22 (PMC9972910; doi:10.1128/mbio.02202-22)
Supplement: TABLE S1 [file mbio.02202-22-s0002.docx]

**Table S1** ***Escherichia coli* strains used in this study**

| **Strain** | **Relevant Characteristics** | | **Reference/Source** |
| --- | --- | --- | --- |
|  | **Chromosomal** | **Plasmid** |  |
| AM604 | MC4100 Ara^+^ |  | (5) |
| DH5α | ∆(*argF*-*lac169*) 80d*lacZ58*(M15) *glnV44*(AS) λ^-^ *rfbD1 gyrA96 recA1 endA1 spoT1 thi-1 hsdR17* |  | (6) |
| KG286.06/pGS404 | *rpsL150* Δ*lptCA* | *ptac*-*lptCA cat* | (7) |
| KG295.01/pGS321 | *rpsL150* Δ*lptCA lptF*^R212G^ | *ptac*-*lptA cat* | (7) |
| KRX | [F’, *tra*D36, Δ*omp*P, *pro*A+B+, *lac*Iq, Δ(*lac*Z)M15] Δ*omp*T, *end*A1, *rec*A1, *gyr*A96, *thi*-1, *hsd*R17 (rK–, mK+), e14– (McrA–), *rel*A1, *sup*E44, Δ(*lac-pro*AB), Δ(*rha*BAD)::T7 gene 1 |  | Promega |
| BL21(DE3) | F^–^ *ompT* *hsdS* (r_B_*^–^*, m_B_^–^) *gal dcm* λ(DE3) |  | Stratagene |
| NEB® 5-alpha | *fhuA2 (argF-lacZ)U169 phoA glnV44 80 (lacZ)*M15 *gyrA96 recA1 relA1 endA1 thi-1 hsdR17* |  | New England Biolabs (NEB) |
| NR1113 | ∆(*λatt lom*)::*bla araBp-lptFG* Δ*lptFG* |  | (8) |
| XL1blue | F^-^ λ^-^ *recA1* *endA1* *gyrA96* *thi-1* *hsdR17* *supE44* *relA1* *lac* F^’^*proAB*, *lacI*q*Z* Δ*M15* Tn*10*(Tet^R^) |  | Agilent Technologies |
| MG1655 | K-12, F^−^ λ^−^ *ilvG^−^ rfb-50 rph-1* |  | (9) |
| C43(DE3) | F^–^ *ompT* *hsdSB* (r_B_*^–^*, m_B_^–^) *gal dcm* λ(DE3) |  | (10) |
