# Supplementary material for: Suppressor Mutations in LptF Bypass Essentiality of LptC by Forming a Six-Protein Transenvelope Bridge That Efficiently Transports Lipopolysaccharide
Source: mBio. 2022 Dec 21;14(1):e02202-22. doi: 10.1128/mbio.02202-22 (PMC9972910; doi:10.1128/mbio.02202-22)
Supplement: TABLE S2 [file mbio.02202-22-s0003.docx]

**Table S2 Plasmids used in this study**

| **Plasmid** | **Relevant Characteristics*^a^*** | **Construction/Origin** |
| --- | --- | --- |
| pBAD/HisA-LptC | pBAD/HisA derivative encoding LptC with replacement of Ser at position 2 to Gly to introduce a NcoI site; Amp^R^ | (11) |
| pCDFDuet-LptB-His_6_FG | pCDFDuet-1 (Novagen) derivative encoding full-length LptB with a C-terminal His_6_ tag, and full-length LptF and LptG; Spn^R^ | (11) |
| pCDFDuet-His_6_-LptBFG | pCDFDuet-LptBHis_6_FG derivative encoding full-length LptB with a N-terminal His_6_ tag, and full-length LptF and LptG | His_6_-LptB was PCR amplified with AP707-AP708 oligos from AM604 genomic DNA and cloned into NcoI-EcoRI sites of pCDFDuet-LptB-His_6_FG replacing *lptB*-His_6_ gene. |
| pCDFDuet-His_6_-LptBF^R212G^G | pCDFDuet-His_6_LptBFG derivative encoding full-length LptB with a N-terminal His_6_ tag, and full-length LptF^R212G^ and LptG | By site-directed mutagenesis using oligos AP613-AP614. |
| pET43.1 Nus-His-LptA | pET43.1b(+) derivative overexpressing NusA-His_6_-LptA | (12) |
| pCOLADuet-LptE-His_6_LptD | pCOLADuet-LptE-His_6_LptD derivative encoding full-length LptD and LptE | LptD was PCR amplified with LptD-pCOLA-fwd and LptD-pCOLA-rev and cloned into pCOLADuet site 2. LptE was PCR amplified LptE-pCOLA-fwd and LptE-pCOLA-rev and cloned into pCOLADuet site 1. |
| pET23/42 | pET23a(+) with multiple cloning sites of pET42a(+), T7 promoter; Amp^R^ | (13) |
| pET23/42-LptB-His | encodes full-length LptB with a C-terminal His_8_ tag | LptB was PCR amplified with AP450 and AP479 from AM604 genomic DNA and cloned into NdeI-XhoI sites of pET23/42. |
| pET23/42-LptC-His | encodes full-length LptC with a C-terminal His_8_ tag | (13) |
| pET23/42-LptF-His | encodes full-length LptF with a C-terminal His_8_ tag | LptF was PCR amplified with FG3295 and FG3296 from AM604 genomic DNA and cloned into NdeI-XhoI sites of pET23/42. |
| pET23/42-LptF^R212G^-His | encodes full-length LptF^R212G^ with a C-terminal His_8_ tag | LptF^R212G^ was PCR amplified with FG3295 and FG3296 from pGS451 and cloned into NdeI-XhoI sites of pET23/42. |
| pET22-42-LptC | encodes full-length LptC with a C-terminal thrombin cleavage site and His_7_ tag | (14)  structural unidirectional basis |
| pEVOL | *aaRS,* tRNACUA opt, p15A origin; Cam^R^ | (2) |
| pEVOL-Spn | pEVOL derivative; Spn^R^ | *aadA* was PCR amplified with AP573 and AP754 from pCDFDuet-1 and cloned into EcoRI site, thus inactivating *cat*. |
| pGS100 | pGZ119EH derivative, contains TIR sequence downstream of *ptac*; Cam^R^ | (15) |
| pGS308 | *ptac*-*lptCA,* *kan, oriV*_ColD_ | (7) |
| pGS323 | *ptac-lptA, kan, oriV*_ColD_ | (7) |
| pGS445 | pGS100 derivative, *ptac- lptFG_lptAB* | (7) |
| pGS451 | pGS100 derivative, *ptac- lptF^R212G^G_lptAB* | (7) |
| pET22b-LptA-I36*p*BPA-His_6_ | pET22b derivative, expresses a C-terminally His tagged LptA with amber mutant at position 36 | (12) |
| pSup- BpaRS-6TRN | *Mj tyrRS,* tRNA _CUA_, p15A origin; Cam^R^ | (16) |
| pQEsH-*lptC* | pQE30 (QIAGEN) derivative, expresses His_6_-LptC_24-191_; Amp^R^ | (17) |
| pET-LptAΔ_160-185_ –H | *pT7-lptAΔ_160-185_* -His_6_; Amp^R^ | (18) |

*^a^* Amp^R^, ampicillin resistance; Spn^R^, spectinomycin resistance; Kan^R^, kanamycin resistance; Cam^R^, chloramphenicol resistance
