# Supplementary material for: Suppressor Mutations in LptF Bypass Essentiality of LptC by Forming a Six-Protein Transenvelope Bridge That Efficiently Transports Lipopolysaccharide
Source: mBio. 2022 Dec 21;14(1):e02202-22. doi: 10.1128/mbio.02202-22 (PMC9972910; doi:10.1128/mbio.02202-22)
Supplement: TABLE S3 [file mbio.02202-22-s0004.docx]

**Table S3 Oligonucleotides used in this study**

| Name | Sequence*^a^* | Notes |
| --- | --- | --- |
| *Plasmid construction* | | |
| AP450 | acacctcgagTCTGAAGTCTTCCCCAAG | pET23/42-LptB-His construction with AP479; XhoI |
| AP479 | acaccatatgGCAACATTAACTGCAAAGAAC | pET23/42 LptB-His construction with AP450; NdeI |
| AP573 | cggaattccgCAGGTATCCGGTAAACGG | pEVOL-Spn construction with AP574; EcoRI |
| AP574 | cggaattccgGCGGCTATTTAACGACCC | pEVOL-Spn construction with AP573; EcoRI |
| AP613 | ACCAGGGAACGGGCTTCGAAGGC | pCDFDuet-His_6_LptBF^R212G^G by site-directed mutagenesis |
| AP614 | TGAGAGTGACGACCTGGGAG | pCDFDuet-His_6_LptBF^R212G^G by site-directed mutagenesis |
| AP707 | tataccatggGCCATCATCATCATCATCACGGAATGGCAACATTAACTGCAAAGAACC | pCDFDuet-His_6_LptBFG construction with AP708; NcoI |
| AP708 | tatagaattcTCAGAGTCTGAAGTCTTCCCCAAGGTATACACG | pCDFDuet-His_6_LptBFG construction with AP707; EcoRI |
| FG3295 | ctggcatatgATAATCATAAGATATCTGGTGCGG | pET23/42 LptF-His and pET23/42 LptF^R212G^-His construction with FG3296; NdeI |
| FG3296 | atatctcgagCACCGCTCCTTTACGCGA | pET23/42 LptF-Hi*s* and pET23/42 LptF^R212G^-Hi*s* construction with FG3295; XhoI |
| pCOLA-site2-fwd | GCAGATCTCAATTGGATATCGGC | pCOLADuet-LptE-His_6_LptD construction to insert LptD with pCOLA-site2-rev |
| pCOLA-site2-rev | ATGTATATCTCCTTCTTATACTTAACTAATATACTAA | pCOLADuet-LptE-His_6_LptD construction to insert LptD with pCOLA-site2-fwd |
| pCOLA-site1-fwd | GCGGCCGCATAATGCTTAAGT | pCOLADuet-LptE-His_6_LptD construction to insert LptE with pCOLA-site1-rev |
| pCOLA-site1-rev | GGTATATCTCCTTATTAAAGTTAAACAAAATTATTTC | pCOLADuet-LptE-His_6_LptD construction to insert LptE with pCOLA-site1-fwd |
| LptD-pCOLA-fwd | GCGGCCGCATAATGCTTAAGT | pCOLADuet-LptE-His_6_LptD construction to insert LptD with LptE-pCOLA-rev |
| LptD-pCOLA-rev | GCCGATATCCAATTGAGATCTGCTCACAAAGTGTTTTGATACGGCAGAAT | pCOLADuet-LptE-His_6_LptD construction to insert LptD with LptE-pCOLA-fwd |
| LptE-pCOLA-fwd | GAAATAATTTTGTTTAACTTTAATAAGGAGATATACCATGGGGCGATATCTGGCAACAT | pCOLADuet-LptE-His_6_LptD construction to insert LptE with LptE-pCOLA-rev |
| LptE-pCOLA-rev | ACTTAAGCATTATGCGGCCGCTCAGTTACCCAGCGTGGTGG | pCOLADuet-LptE-His_6_LptD construction to insert LptE with LptE-pCOLA-fwd |
| *Generation of LptF amber mutants* | | |
| AP500 | CACTCTCAACCAGGGAACGtagTTCGAAGGCACTGCATTG | LptF amber mutant generation with AP501; **R212am** |
| AP501 | CAATGCAGTGCCTTCGAActaCGTTCCCTGGTTGAGAGTG | LptF amber mutant generation with AP500; **R212am** |
| AP512 | TGGCAGCTCGGTGCTGtagATCGAAAGCGTTGACG | LptF and LptF^R212G^ amber mutant generation with AP513; **F160am** |
| AP513 | CGTCAACGCTTTCGATctaCAGCACCGAGCTGCCA | LptF and LptF^R212G^ amber mutant generation with AP512; **F160am** |
| AP514 | CTGCGCGACGGCTCCtagGTCGTCACTCTCAAC | LptF and LptF^R212G^ amber mutant generation with AP515; **Q203am** |
| AP515 | GTTGAGAGTGACGACctaGGAGCCGTCGCGCAG | LptF and LptF^R212G^ amber mutant generation with AP514; **Q203am** |
| AP518 | ACCAGGGAACGCGCTTCtagGGCACTGCATTGTTACG | LptF amber mutant generation with AP519; **E214am** |
| AP519 | CGTAACAATGCAGTGCCctaGAAGCGCGTTCCCTGGT | LptF amber mutant generation with AP518; **E214am** |
| AP524 | CTCAACCAGGGAACGGGCtagGAAGGCACTGCATTGTTAC | LptF^R212G^ amber mutant generation with AP525; **F213am** |
| AP525 | GTAACAATGCAGTGCCTTCctaGCCCGTTCCCTGGTTGAG | LptF^R212G^ amber mutant generation with AP524; **F213am** |
| AP603 | AACGGGCTTCtagGGCACTGCAT | LptF^R212G^ amber mutant generation with AP604; **E214am** |
| AP604 | CCCTGGTTGAGAGTGACG | LptF^R212G^ amber mutant generation with AP603; **E214am** |
| AP605 | CTTCCAGGATtagCAGGCGATCA | LptF and LptF^R212G^ amber mutant generation with AP606; **Y230am** |
| AP606 | TCCGTAATGCGGAAATCAC | LptF and LptF^R212G^ amber mutant generation with AP605; **Y230am** |
| *^a^* Upper case letters, sequence present in the template; lower case letters, additional/modified sequence not present in the template; restriction sites are underlined. | | |
