# Supplementary material for: Suppressor Mutations in LptF Bypass Essentiality of LptC by Forming a Six-Protein Transenvelope Bridge That Efficiently Transports Lipopolysaccharide
Source: mBio. 2022 Dec 21;14(1):e02202-22. doi: 10.1128/mbio.02202-22 (PMC9972910; doi:10.1128/mbio.02202-22)
Supplement: TEXT S1 [file mbio.02202-22-s0001.pdf]

SUPPLEMENTAL MATERIAL

Suppressor mutations in LptF bypass essentiality of LptC by forming a six-protein trans-envelope bridge that efficiently transports lipopolysaccharide

Federica A. Falchi[a*], Rebecca J. Taylor[b], Sebastian J. Rowe[b], Elisabete C. C. M. Moura[a*], Tiago Baeta[c], Cedric Laguri[c], Jean-Pierre Simorre[c], Daniel E. Kahne[b,e], Alessandra Polissi[a], Paola Sperandeo[a#].

[a] Dipartimento di Scienze Farmacologiche e Biomolecolari, Università degli Studi di Milano, Milan, Italy

[b]Department of Chemistry and Chemical Biology, Harvard University, Cambridge, MA, USA

[c]University of Grenoble Alpes, CNRS, CEA, IBS, Grenoble, France

[e]Department of Biological Chemistry and Molecular Pharmacology, Harvard Medical School, Boston, MA, USA

Running Head: Control of LPS transport in Gram-negative bacteria

#Address correspondence to: paola.sperandeo@unimi.it

[*] Present address: Federica A. Falchi, Dipartimento di Bioscienze, Università degli Studi di Milano, Milan, Italy;

[*] Present address: Elisabete C. C. M. Moura, Olgram SAS, Brehan, France.

F.A.F and R.J.T contributed equally to this work. The order of authors was decided by mutual agreement.

S.J.R, E.C.C.M. and T.B. contributed equally to this work.

INDEX

Text S1. Supplemental methods.

Table S1. *Escherichia coli* strains used in this study.

Table S2. Plasmids used in this study.

Table S3. Oligonucleotides used in this study.

Figure S1. LptBF^{R212G}G does not prevent interaction with LptC.

Figure S2. Position of residues in LptB$_2$FGC substituted with amber codons.

Figure S3. Mutant LptF^{R212G} interacts with LptA and interaction is lost in the presence of ectopically expressed LptC.

Figure S4. Determination of the dissociation constants of the interactions LptB$_2$FG-LptAm and LptB$_2$F^{R212G}G-LptAm by SPR.

Figure S5. Gating strategy for the collection of the FACS data.

SUPPLEMENTAL METHODS

***In vivo* site-specific UV-photocrosslinking and whole-cell lysate analysis for LptF-LptA interaction.** Amber codons were introduced into pGS445 and pGS451 plasmids, harbouring *lptFGAB* and *lptF^{R212G}GAB*, respectively, pEVOL-Spn vector (1, 2) was used to incorporate *p*-benzoyl-L-phenylalanine (*p*Bpa) into LptF or LptF^{R212G}. Plasmids carrying amber mutations were used to transform AM604 strain. When indicated, cells were also transformed with pBAD/HisA-LptC plasmid to overexpress LptC. Diploid strains were used for all experiments and the protocol was modified from Simpson *et al*. (3). Briefly, overnight cultures were diluted in 10 mL of LB medium containing 0.45 mM *p*BPA (Bachem) and 0.2% arabinose to an optical density at 600 nm (OD_{600}) ~ 0.1. Strains were grown to OD_{600} ~ 0.5 – 0.7. A total amount of 1 OD of cells was transferred to a 6-well flat-bottom cell culture plate (Greiner bio-one) and irradiated for 10 min on ice with a UV lamp (365 nm). An additional sample corresponding to 1 OD of cells was used as UV-untreated control. Samples were then TCA precipitated (10% w/v trichloroacetic acid final), resuspended in 100 µl of sample buffer SDS sample buffer for SDS−PAGE and analysed by western blotting using anti-LptA and anti-LptF antibodies.

ATPase activity assay on DDM-solubilized complexes. The ATPase activity of 0.2 µM $LptB_2FG$ and $LptB_2F^{R212G}G$ purified complexes, or 0.1 µM $LptB_2FGC$ and $LptB_2F^{R212G}GC$ purified complexes, was measured in 50 mM Tris-HCl, pH 8.0, 500 mM NaCl, 10% glycerol, 0.01% DDM. The reaction was started at room temperature by the addition of 5 mM ATP/$MgCl_2$. Aliquots (15 µL) were taken at 0, 5, 10, 20, 40, and 60 min and mixed with the same volume of 12% SDS to stop the reaction. The amounts of inorganic phosphate were determined according to the method reported in (4). Absorbance was read at 850 nm using the EnSpire Multimode Plate Reader (PerkinElmer). The assay was repeated three times for each condition and the results were analysed with GraphPad Prism

(GraphPad Software, Inc., San Diego, CA, USA). Plotted data represents the mean value of 3 replicates, error bars indicate the standard deviation.

Surface Plasmon Resonance (SPR)-based binding assay. $LptB_2FG$ and $LptB_2F^{R212G}G$ purified in DDM were immobilized onto a CM5 chip in a T200 instrument (GE Healthcare) through standard EDC/NHS method in 10 mM MES pH 6.0, 300 µM DDM at 21°C. About 4000 RU of immobilized protein was achieved. LptAm was injected at different concentrations in 20 mM Tris, 150 mM NaCl, 300 µM DDM, pH 8.0 buffer. The sensorchip was regenerated between two injections with 10 s NaOH 10 mM, 300 µM DDM pulses. Sensorgrams accounting from buffer alone and from a control sensorchip were subtracted prior to analysis. Kd was determined with the BiaEval software considering steady state binding prior to dissociation.

SUPPLEMENTAL REFERENCES

1. Cho SH, Szewczyk J, Pesavento C, Zietek M, Banzhaf M, Roszczenko P, Asmar A, Laloux G, Hov AK, Leverrier P, Van der Henst C, Vertommen D, Typas A, Collet JF. 2014. Detecting envelope stress by monitoring beta-barrel assembly. Cell 159:1652-64.
2. Young TS, Ahmad I, Yin JA, Schultz PG. 2010. An enhanced system for unnatural amino acid mutagenesis in *E. coli*. J Mol Biol 395:361-74.
3. Simpson BW, Owens TW, Orabella MJ, Davis RM, May JM, Trauger SA, Kahne D, Ruiz N. 2016. Identification of Residues in the Lipopolysaccharide ABC Transporter That Coordinate ATPase Activity with Extractor Function. mBio 7.
4. Chifflet S, Torriglia A, Chiesa R, Tolosa S. 1988. A method for the determination of inorganic phosphate in the presence of labile organic phosphate and high concentrations of protein: application to lens ATPases. Anal Biochem 168:1-4.
5. Wu T, McCandlish AC, Gronenberg LS, Chng SS, Silhavy TJ, Kahne D. 2006. Identification of a protein complex that assembles lipopolysaccharide in the outer membrane of *Escherichia coli*. Proc Natl Acad Sci U S A 103:11754-9.
6. Hanahan D. 1983. Studies on transformation of Escherichia coli with plasmids. J Mol Biol 166:557-80.
7. Benedet M, Falchi FA, Puccio S, Di Benedetto C, Peano C, Polissi A, Deho G. 2016. The Lack of the Essential LptC Protein in the Trans-Envelope Lipopolysaccharide Transport Machine Is Circumvented by Suppressor Mutations in LptF, an Inner Membrane Component of the *Escherichia coli* Transporter. PLoS One 11:e0161354.
8. Ruiz N, Gronenberg LS, Kahne D, Silhavy TJ. 2008. Identification of two inner-membrane proteins required for the transport of lipopolysaccharide to the outer membrane of *Escherichia coli*. Proc Natl Acad Sci U S A 105:5537-42.
9. Blattner FR, Plunkett G, 3rd, Bloch CA, Perna NT, Burland V, Riley M, Collado-Vides J, Glasner JD, Rode CK, Mayhew GF, Gregor J, Davis NW, Kirkpatrick HA, Goeden MA,

Rose DJ, Mau B, Shao Y. 1997. The complete genome sequence of *Escherichia coli* K-12. Science 277:1453-62.

10. Dumon-Seignovert L, Cariot G, Vuillard L. 2004. The toxicity of recombinant proteins in *Escherichia coli*: a comparison of overexpression in BL21(DE3), C41(DE3), and C43(DE3). Protein Expr Purif 37:203-6.
11. Narita S, Tokuda H. 2009. Biochemical characterization of an ABC transporter LptBFGC complex required for the outer membrane sorting of lipopolysaccharides. FEBS Lett 583:2160-4.
12. Sherman DJ, Xie R, Taylor RJ, George AH, Okuda S, Foster PJ, Needleman DJ, Kahne D. 2018. Lipopolysaccharide is transported to the cell surface by a membrane-to-membrane protein bridge. Science 359:798-801.
13. Chng SS, Gronenberg LS, Kahne D. 2010. Proteins required for lipopolysaccharide assembly in *Escherichia coli* form a transenvelope complex. Biochemistry 49:4565-7.
14. Owens TW, Taylor RJ, Pahil KS, Bertani BR, Ruiz N, Kruse AC, Kahne D. 2019. Structural basis of unidirectional export of lipopolysaccharide to the cell surface. Nature 567:550-553.
15. Sperandeo P, Pozzi C, Deho G, Polissi A. 2006. Non-essential KDO biosynthesis and new essential cell envelope biogenesis genes in the *Escherichia coli yrbG-yhbG* locus. Res Microbiol 157:547-58.
16. Ryu Y, Schultz PG. 2006. Efficient incorporation of unnatural amino acids into proteins in *Escherichia coli.* Nat Methods 3:263-5.
17. Sperandeo P, Villa R, Martorana AM, Samalikova M, Grandori R, Deho G, Polissi A. 2011. New insights into the Lpt machinery for lipopolysaccharide transport to the cell surface: LptA-LptC interaction and LptA stability as sensors of a properly assembled transenvelope complex. J Bacteriol 193:1042-53.
18. Laguri C, Sperandeo P, Pounot K, Ayala I, Silipo A, Bougault CM, Molinaro A, Polissi A, Simorre JP. 2017. Interaction of lipopolysaccharides at intermolecular sites of the periplasmic Lpt transport assembly. Sci Rep 7:9715.
